# Rosavin Alleviates LPS-Induced Acute Lung Injure by Modulating the TLR-4/NF-κB/MAPK Singnaling Pathways

**DOI:** 10.3390/ijms25031875

**Published:** 2024-02-03

**Authors:** Qiao-Hui Liu, Ke Zhang, Shu-Shu Feng, Li-Juan Zhang, Shun-Ying Li, Hang-Yu Wang, Jin-Hui Wang

**Affiliations:** 1 Key Laboratory of Xinjiang Phytomedicine Resource and Utilization, Ministry of Education, College of Pharmacy, Shihezi University, Shihezi 832002, Chinawangjinhui@hrbmu.edu.cn (J.-H.W.); 2 State-Province Key Laboratory of Biomedicine-Pharmaceutics of China, Department of Medicinal Chemistry and Natural Medicine Chemistry, Harbin Medical University, Harbin 150081, China

**Keywords:** rosavin, acute lung injury, anti-inflammatory, TLR-4/NF-κB/MAPK pathway, network pharmacology, transcriptome sequencing

## Abstract

Acute lung injury (ALI) is a serious inflammatory disease with high morbidity and mortality. Rosavin is an anti-inflammatory and antioxidant phenylpropanoid and glucoside, which is isolated from Rhodiola rosea L. However, its potential molecular mechanisms and whether it has protective effects against lipopolysaccharide (LPS)-induced ALI remain to be elucidated. To assess the in vitro anti-inflammatory effects and anti-lung injury activity of rosavin, RAW264.7 and A549 cells were stimulated using 1 μg/mL LPS. Rosavin attenuated LPS-induced activation of the TLR-4/NF-κB signaling pathway in RAW264.7 cells and inhibited LPS-induced release of inflammatory factors in A549 cells. A mouse model of acute lung injury was constructed by intraperitoneal injection of 5 mg/kg LPS to observe the therapeutic effect of rosavin. Transcriptomics analysis and Western blot assays were utilized to verify the molecular mechanism, rosavin (20, 40, and 80 mg/kg) dose-dependently ameliorated histopathological alterations, reduced the levels of inflammatory factors, and inhibited the TLR-4/NF-κB/MAPK signaling pathway and apoptosis activation. Rosavin is a promising therapeutic candidate for acute lung injury by inhibiting the TLR-4/NF-κB/MAPK pathway.

## 1. Introduction

Acute lung injury (ALI) is a severe respiratory disease characterized by rapid alveolar injury, severe hypoxemia, an uncontrolled inflammatory response, and cytokine accumulation, which can progress to acute respiratory distress syndrome (ARDS) [1]. In recent years, conventional anti-inflammatory drug therapy and mechanical ventilation have provided some relief from the stress of lung injury; however, ALI remains a serious disease with high mortality and morbidity [2,3]; the development of new and effective treatments for ALI is required to limit excessive inflammation and to improve the status of treatment for ALI [4,5].

Acute lung injury begins with uncontrolled inflammatory responses that are initiated, amplified, and modulated by the high levels of reactive oxygen species (ROS) produced by oxidative stress, along with various pro-inflammatory molecules and cytokines produced by multiple inflammatory cells [6,7]. Pathogen-associated molecular patterns (PAMPs), such as lipopolysaccharide (LPS), can bind the cell surface Toll like receptor (TLR-4) and subsequently stimulate the recruitment and activation of medullary differentiation major response gene 88 (MyD88) [8,9], which in turn, leads to the activation of intracellular pathways such as nuclear factor -kappa b (NF-κB). NF-κB is a multi-directional, omnipotent regulator that is central to the inflammatory response [8,9,10]. p38 MAPK is also an important kinase for TLR-4/MyD88 activation, as well as for the transcription of numerous important pro-inflammatory factors [11], including tumor necrosis factor (TNF-α), interleukin 6 (IL-6), and interleukin 1β (IL-1β) and nitric oxide (NO) [12]. Lung injury induced by the intraperitoneal injection of LPS is one of the more common models of lung injury; the disadvantages of this induction of ALI/ARDS are the long time required to cause lung injury and the involvement of other organ systems; however, the model is highly reproducible and simulates the inflammatory response of ALI/ARDS [13,14]. Meanwhile, it has been demonstrated that A549 cells are already sensitive to 1 μg/mL of LPS, and can be stimulated to release inflammatory factors [15,16], whereas in the RAW264.7 cell line, this concentration already promotes the activation of signaling pathways such as mitogen-activated protein kinase (MAPK) [17,18]. LPS is a key factor in the induction of acute respiratory infection [19,20]. Therefore, intervening with TLR-4/NF-κB/MAPK-mediated inflammatory responses may be a feasible strategy for treating LPS-induced ALI.

Natural products are important resources for drug development and utilization research [21,22,23]. *Rhodiola rosea* L is a medicinal herb commonly used to treat altitude sickness (mountain hypoxia) and hypoxia [24,25]. Rosavin is a phenylpropanoid and its glucoside was isolated from *Rhodiola rosea* L. It attenuates sepsis-induced lung injury by inhibiting neutrophil extracellular trap formation; its inhibitory effect may be exerted through the modulation of the MAPK pathway [26]. It also prevents (particulate matter) PM2.5-stimulated lung injury by up-regulating the PI3K/Akt/Nrf2 signaling pathway [27]. It has a protective effect on bleomycin-induced pulmonary fibrosis in mice, and the underlying mechanism may be related to the inhibition of the inflammatory response [28]. However, further investigation is required to determine whether rosavin has beneficial effects on LPS-induced ALI.

In this study, based on the in vivo and in vitro LPS-induced ALI model, we found that rosavin was able to eliminate lung inflammation by inhibiting the activation of the TLR-4/NF-κB/MAPK signaling pathway and apoptosis, which not only provides theoretical ideas for elucidating the possible molecular mechanism of rosavin but also provides a basis for the potential clinical application of rosavin in the future treatment of ALI.

## 2. Results

### 2.1. Prediction and Analysis of Potential Targets for Rosavin and ALI

**Figure 1:**
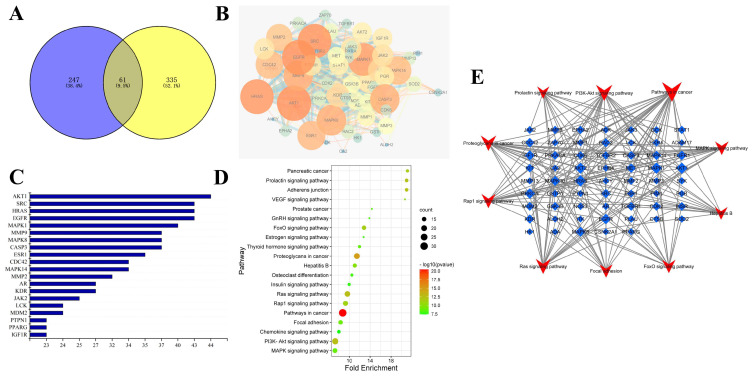
Network pharmacological analysis of rosavin and ALI. (**A**) Venn analysis of rosavin and ALI targets. (**B**) Protein network interactions. (**C**) Histogram of top 20 genes acted on by PPI. (**D**) KEGG enrichment bubble diagram. (**E**) Target-pathway diagram.

A total of 308 targets for rosavin were screened using the Swiss Target Prediction platform, and 396 targets for ALI were screened using GeneCards and OMIM databases. A total of 61 potential targets for the prevention of ALI by rosavin were obtained from Venn analysis (Figure 1A). The top 20 key targets were screened for AKT1, SRC, HRAS, EGFR and MAPK1 (Figure 1B,C) using the STRING database to analyze the relevant protein network interactions. The top 20 pathways were screened using KEGG pathway enrichment analysis (Figure 1D); they included the MAPK, PI3K-Akt, Rap 1 and Ras signaling pathways. The target pathways were subsequently mapped (Figure 1E). This suggests that rosavin may play a role in the treatment of ALI through these pathways.

### 2.2. In Vitro Results

#### 2.2.1. Rosavin Inhibits Pro-Inflammatory Mediator Release and Oxidative Stress Generated by LPS in RAW264.7 Cells

**Figure 2:**
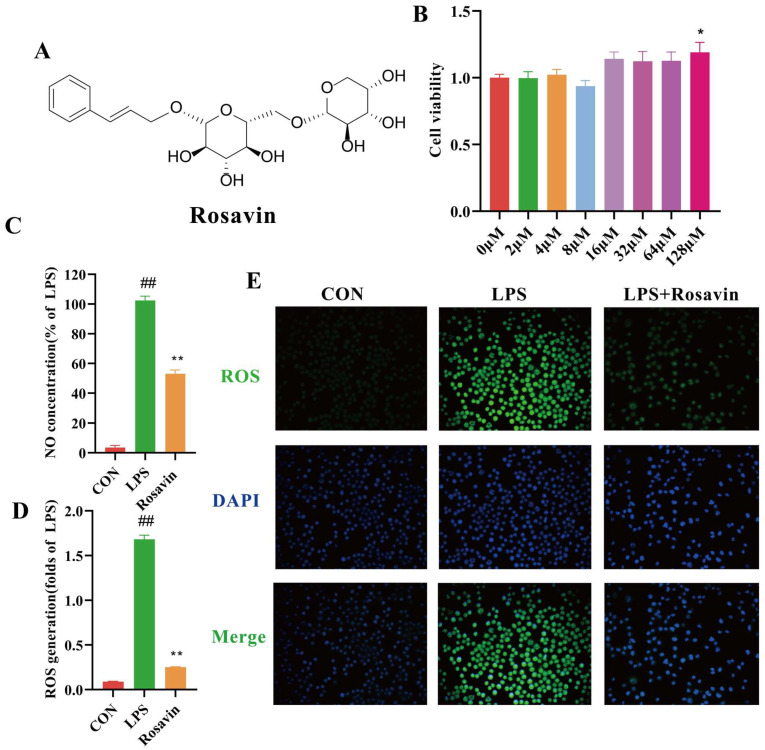
Rosavin inhibits pro-inflammatory mediator release and oxidative stress generated by LPS in RAW264.7 cells. RAW264.7 cells were treated with 64 μM rosavin and LPS (1 μg/mL) for 24 h. (**A**) Chemical structure of rosavin. (**B**) Effect of rosavin on the viability of RAW264.7 cells. (**C**) Measurement of NO content. (**D**) ROS measurements by microplate reader. (**E**) ROS measurements by fluorescence microscope. Scale bar = 50 μm. Data are expressed as the mean ± standard deviation (*n* = 3); ^##^ *p* < 0.01 vs. control group; * *p* < 0.05, ** *p* < 0.01 vs. LPS group.

We performed the relevant MTT experiments to determine whether rosavin affects the viability of RAW264.7 (Figure 2B). Rosavin had no significant effect on RAW264.7 cell viability up to 128 μM; therefore, we chose concentrations of 16, 32, and 64 μM for the subsequent experiments. The influence of rosavin on the production of the pro-inflammatory mediator NO was studied to assess its possible anti-inflammatory capabilities. Lipopolysaccharide drastically enhanced NO release from RAW264.7 cells (*p* < 0.01) (Figure 2C). However, rosavin reduced the stimulating effect of 1 μg/mL LPS (*p* < 0.01). Furthermore, the development of inflammation typically leads to an overabundance of ROS. Cells were stained with DCFH-DA to investigate the effect of rosavin on oxidative stress and to determine intracellular ROS levels. As expected, LPS treatment increased green fluorescence (*p* < 0.01), whereas rosavin treatment decreased intracellular ROS production, according to fluorescence microscopy and the fluorescence intensity assay (*p* < 0.01) (Figure 2D,E).

#### 2.2.2. Rosavin Inhibits the TLR-4/NF-κB Signaling Cascade Generated by LPS in RAW264.7 Cells

**Figure 3:**
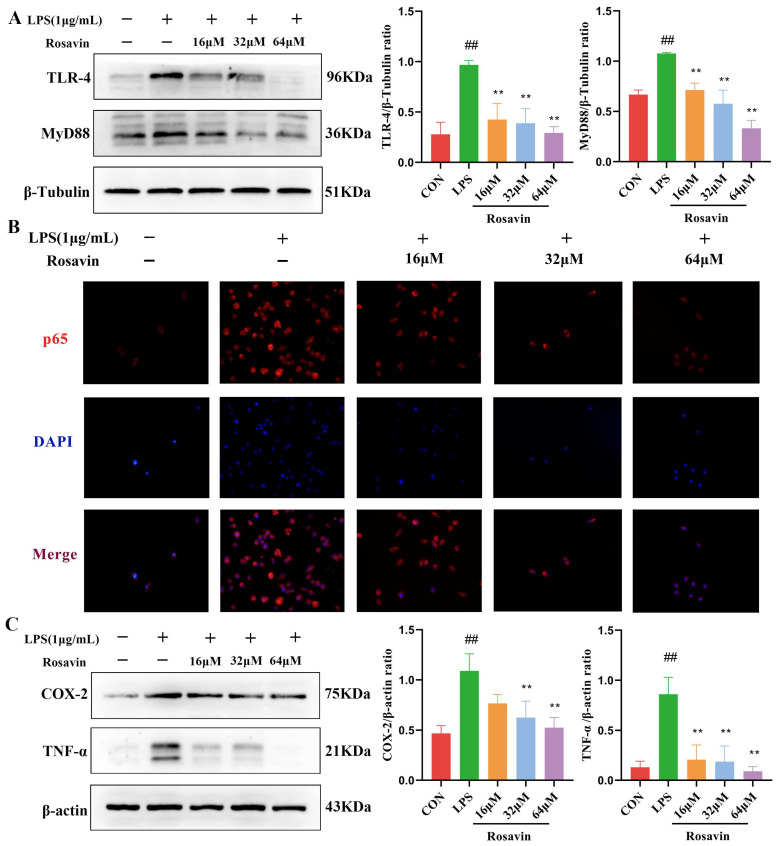
Rosavin inhibits the TLR-4/NF-κB signaling cascade generated by LPS in RAW264.7 cells. RAW264.7 cells were treated with 0, 16, 32, 64 μM rosavin and LPS (1 μg/mL) for 24 h. (**A**) Relative protein levels of TLR-4 and MyD88 in RAW264.7 cells. (**B**) Subcellular localization of NF-κB p65 in LPS-stimulated RAW264.7 cells. Scale bar = 50 μm. (**C**) Relative protein levels of COX-2 and TNF-α in RAW264.7 cells. Data are expressed as the mean ± standard deviation (*n* = 3); ^##^ *p* < 0.01 vs. control group; ** *p* < 0.01 vs. LPS group.

Lipopolysaccharide binds TLR-4 and activates the downstream NF-κB pathway, releasing pro-inflammatory cytokines at the site of tissue damage. We quantified the activation of the TLR-4/NF-κB to further investigate the protective effect of rosavin against LPS-induced inflammation. Western blotting results showed that LPS stimulated the activation of TLR-4 protein in RAW264.7 cells, and rosavin markedly reversed this effect (*p* < 0.01) (Figure 3A). NF-κB p65 is an important protein involved in the inflammatory response. Immunofluorescence images showed that more p65 was located in the nucleus of the cells after LPS stimulation of RAW264.7 cells, and rosavin markedly reversed this effect (*p* < 0.01) (Figure 3B). COX-2 catalyzes the production of PGE2, and TNF-α is a pro-inflammatory cytokine; both proteins promote the development of inflammation and are indicators of inflammation following NF-κB pathway activation. Lipopolysaccharide stimulated increased levels of TNF-α and COX-2 proteins, which were significantly reversed by rosavin treatment in a dose-dependent manner (Figure 3C). Thus, rosavin inhibited the LPS-induced TLR-4/NF-κB signaling cascade in vitro.

#### 2.2.3. Rosavin Attenuated the Inflammation Response Cascade of LPS-Induced in A549 Cells

**Figure 4:**
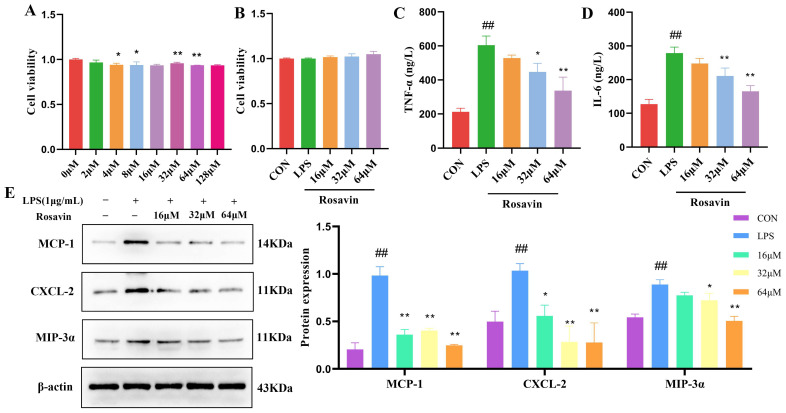
Rosavin attenuated the inflammation response cascade of LPS-induced in A549 cells. A549 cells were treated with 0, 16, 32, 64 μM rosavin and LPS (1 μg/mL) for 24 h. (**A**,**B**) Effect of rosavin on the viability. (**C**) Measurement of TNF-α content. (**D**) Measurement of IL-6 content. (**E**) Effect of rosavin on MCP-1, CXCL-2, MIP-3α protein expression. Data are shown as the mean ± standard deviation (*n* = 3); ^##^ *p* < 0.01 vs. control group; * *p* < 0.05, ** *p* < 0.01 vs. LPS group.

Rosavin was not toxic to A549 cells at concentrations up to 128 μM according to an MTT assay (Figure 4A). Therefore, we chose 16, 32, and 64 μM as the stimulating concentrations for in vitro experiments (Figure 4B). The release of inflammatory mediators is an important factor that contributes to the development of lung injury. The release of pro-inflammatory factors IL-6 and TNF-α in the LPS group significantly increased (*p* < 0.01), and the release of pro-inflammatory factors in the supernatant of all rosavin dose groups decreased to varying degrees (Figure 4C,D) (*p* < 0.01) compared with the control group, the expression of pro-inflammatory mediators such as macrophage chemotactic protein (MCP-1), chemokine (C-X-C Motif) ligand 2 (CXCL2), and macrophage inflammatory protein-3α (MIP-3α) was significantly increased after LPS treatment, and the expression of these proteins decreased to different degrees after rosavin treatment (Figure 4E) (*p* < 0.01).

#### 2.2.4. Rosavin Inhibits LPS-Induced Activation of the MAPK Signaling Pathway in A549 Cells

**Figure 5:**
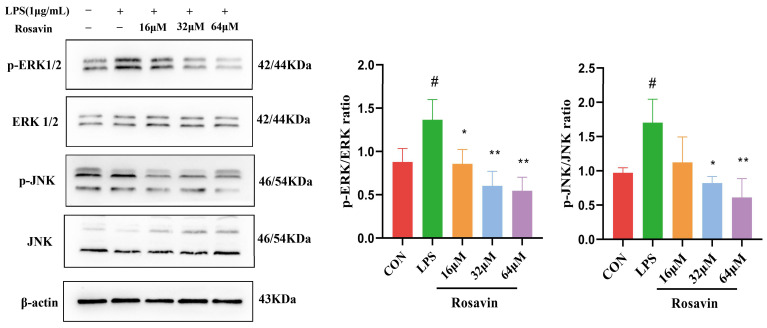
Effect of rosavin on p-ERK and p-p38 protein expression in A549 cells. A549 cells were treated with 0, 16, 32, 64 μM rosavin and LPS (1 μg/mL) for 24 h. Data are shown as the mean ± standard deviation (*n* = 3); ^#^ *p* < 0.05 vs. control group; * *p* < 0.05, ** *p* < 0.01 vs. LPS group.

We measured the expression of MAPK proteins (ERK1/2, JNK and their phosphorylated versions) in A549 cells. Lipopolysaccharide substantially enhanced MAPK phosphorylation compared with the control group (*p <* 0.01). Meanwhile, the phosphorylation of ERK1/2, JNK, and p38 was substantially lower in the rosavin-treated group (*p* < 0.05) (Figure 5).

#### 2.2.5. Rosavin Suppresses LPS-Induced Apoptosis in A549 Cells

**Figure 6:**
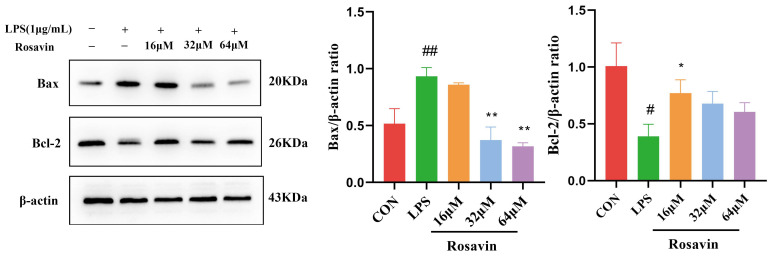
Effect of rosavin on Bax and Bcl-2 protein expression in A549 cells. A549 cells were treated with 0, 16, 32, 64 μM rosavin and LPS (1 μg/mL) for 24 h. Data are shown as the mean ± standard deviation (*n* = 3); ^#^ *p* < 0.05, ^##^ *p* < 0.01 vs. control group; * *p* < 0.05, ** *p* < 0.01 vs. LPS group.

We quantified the expression levels of the relevant proteins in A549 cells. Lipopolysaccharide treatment increased the expression of the pro-apoptotic protein Bax and decreased the expression of the anti-apoptotic protein Bcl-2, compared with the control group (*p* < 0.01). Lipopolysaccharide-induced apoptosis was greatly attenuated in the rosavin-treated group (*p* < 0.05) (Figure 6).

### 2.3. In Vivo Results

#### 2.3.1. Rosavin Protects Mice from ALI Induced by LPS

**Figure 7:**
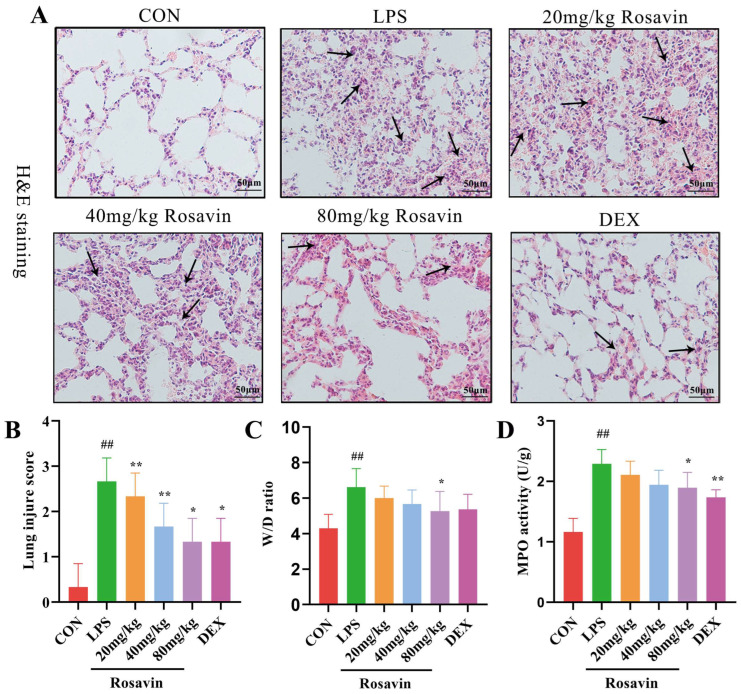
Rosavin protects mice from LPS-induced acute lung injury. (**A**) HE staining results of lung tissue (scale bar = 50 μm, black arrows indicate inflammatory infiltration). (**B**) Lung injury score. (**C**) W/D of lung tissue. (**D**) Myeloperoxidase activity of lung tissue. Data are expressed as the mean ± standard deviation (*n* = 6); ^##^ *p* < 0.01 vs. control group; * *p* < 0.05, ** *p* < 0.01 vs. LPS group.

**Figure 8:**
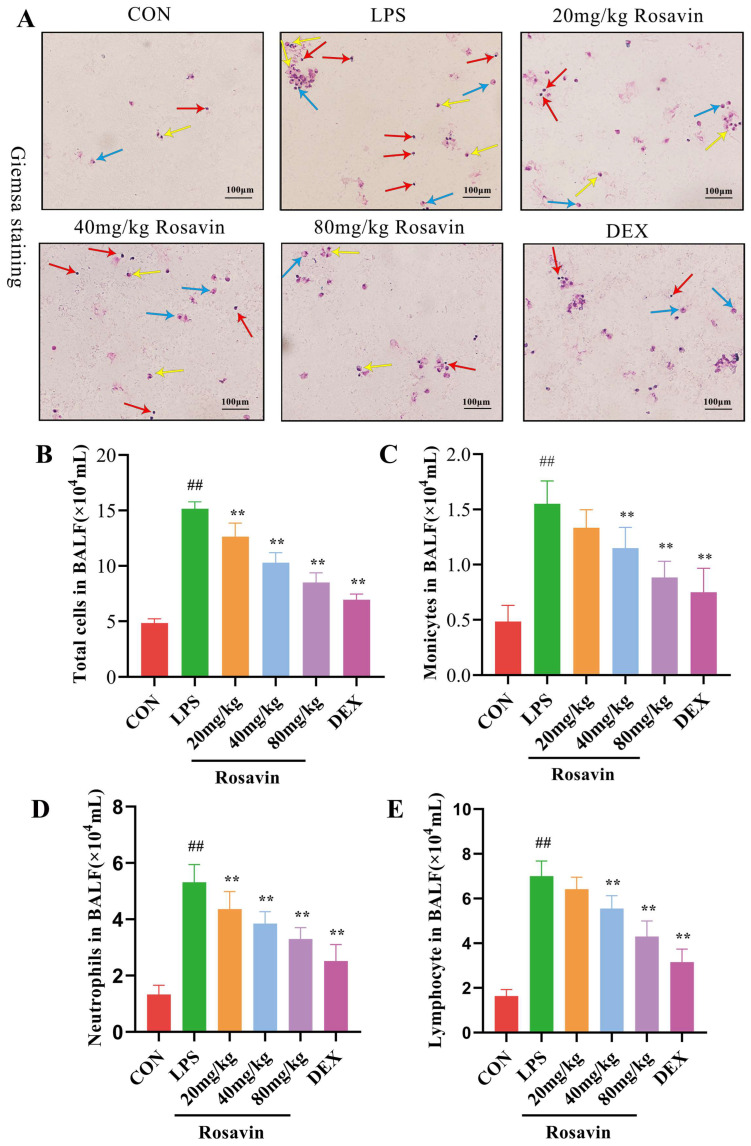
Rosavin protects mice from LPS-induced acute lung injury. (**A**) Micrographs of Giemsa staining of lung tissue (scale bar = 100 μm, blue arrows indicate monocytes, yellow arrows indicate neutrophils, red arrows indicate lymphocytes). (**B**) Total cell count results in alveolar lavage fluid from each group of mice. (**C**) Monocyte count in alveolar lavage fluid. (**D**) Neutrophil count in alveolar lavage fluid. (**E**) Lymphocyte count in alveolar lavage fluid. Data are expressed as the mean ± standard deviation (*n* = 6); ^##^ *p* < 0.01 vs. control group; ** *p* < 0.01 vs. LPS group.

Based on the preliminary in vitro activity, a mouse model of LPS-induced acute lung damage was developed to further investigate the preventive characteristics of rosavin against lung injury in vivo. The lung tissue structure in the control group was adequately observed under a light microscope, with intact and identifiable alveolar structures, no signs of edema in the interstitial area, and no lymphocyte exudation. Meanwhile, lung sections from mice in the LPS group showed extensive morphological alterations, substantial deterioration of the pulmonary structure, diffuse gridlock, fluid retention in the interstitial space, and a large number of neutrophils in the alveolar cavity (Figure 7A,B) according to H&E staining and inflammatory scoring of the lung tissue sections. Rosavin and DEX administration improved pathological changes, including neutrophil infiltration into the interstitial space, thickening of the alveolar septa, and reduced lung injury grade. The wet/dry proportion of the lung tissue indirectly reflects lung edema. Lung W/D values were substantially higher in the LPS group (*p <* 0.01); however, treatment with 80 mg/kg rosavin markedly diminished lung edema compared with the LPS group (*p <* 0.05) (Figure 7C). Myeloperoxidase is mostly found in neutrophils; therefore, measuring its activity in the lung tissue is a helpful predictor of neutrophil increase. Its secretion was drastically augmented in the LPS group (*p* < 0.01) (Figure 7D). MPO concentration in the rosavin intervention group decreased to varying degrees compared with the LPS group, and the administration of 80 mg/kg rosavin significantly lowered MPO activity (*p* < 0.01). Furthermore, the development of acute lung damage is frequently accompanied by the infiltration of inflammatory cells (such as neutrophils, lymphocytes, and monocytes). Inflammatory cell counts in the alveolar lavage fluid were noticeably elevated in the LPS group (Figure 8A–E) (*p* < 0.01); inflammatory cell counts in the alveolar lavage fluid were remarkably diminished in the DEX group compared with the LPS group (*p* < 0.01), and inflammatory cell counts were dose-dependently reduced by the different doses of rosavin (*p* < 0.05).

#### 2.3.2. Rosavin Ameliorates LPS-Induced Release of Inflammatory Mediators and Oxidative Stress Injury in Mice

**Figure 9:**
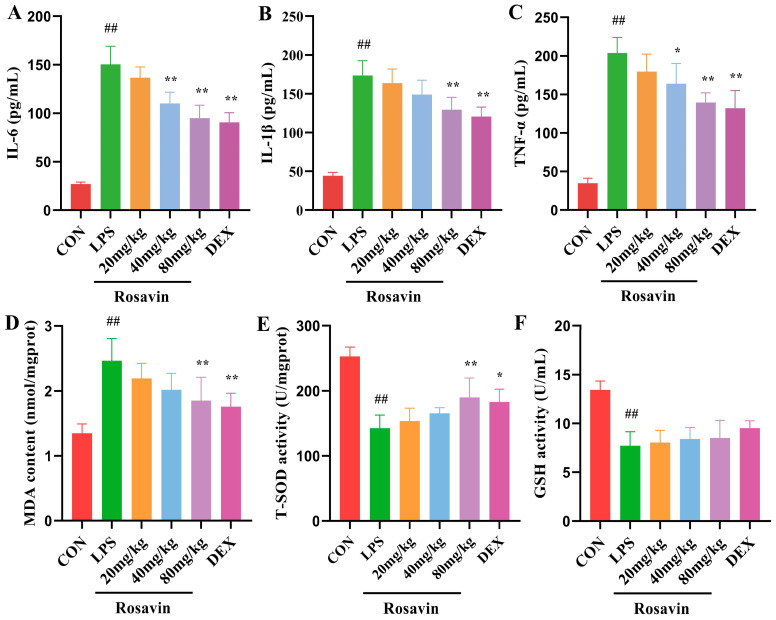
Effect of rosavin on BALF liberation of IL-6, IL-1β and TNF-α and the effect of rosavin on MDA, T-SOD, and GSH activity in lung tissues. (**A**) IL-6 concentration. (**B**) IL-1β concentration. (**C**) TNF-α concentration. (**D**) MDA content. (**E**) T-SOD activity. (**F**) GSH activity. Data are shown as the mean ± standard deviation (*n* = 6); ^##^ *p* < 0.01 vs. control group; * *p* < 0.05, ** *p* < 0.01 vs. LPS group.

The release of inflammatory mediators significantly contributes to the development of lung damage. We used ELISA to detect the levels of IL-6, IL-1β, and TNF-α in mouse BALF (Figure 9A–C). The liberation of pro-inflammatory factors IL-6, IL-1β, and TNF-α was substantially augmented in the LPS group (*p* < 0.01). Compared with the LPS group, the liberation of pro-inflammatory factors in the BALF of all rosavin high-dose groups declined differentially (*p* < 0.01). Finally, rosavin drastically reduced the LPS-induced inflammatory cascade in the mouse lungs. Malondialdehyde is one of the indexes commonly used to estimate the degree of oxidative stress, and SOD and GSH are naturally occurring superoxide radical scavenging factors in the organism. The MDA content of the LPS group considerably increased (Figure 9D–F) compared with the control group (*p* < 0.01), whereas the SOD content and GSH activity drastically declined (*p* < 0.01). The MDA value of the rosavin dosage groups drastically decreased compared with the LPS group, whereas the SOD content and GSH activity drastically increased (*p* < 0.05). Rosavin affects the oxidative and antioxidant balance in mouse lung tissue, which helps to reduce LPS-induced acute lung damage.

#### 2.3.3. Transcriptomic Analysis

**Figure 10:**
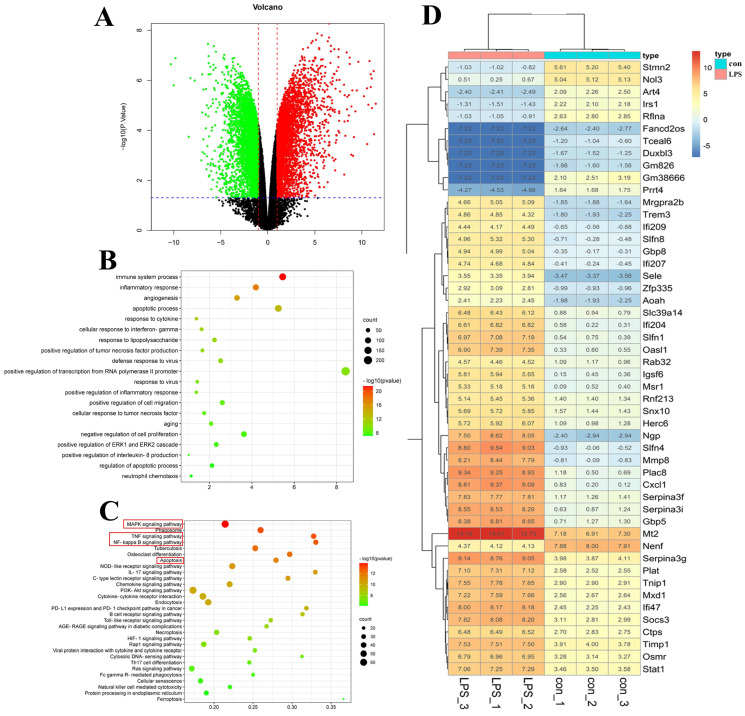
Transcriptomics analysis results of the control group and LPS group. (**A**) Volcano graph. Black dots represent genes, red dots represent up-regulated genes, green dots represent down-regulated genes, and the red line represents the screening screening condition of |log_2_FC| > 1, and the blue line represents the screening condition of *p* < 0.05. (**B**) GO biological process enrichment graph. (**C**) KEGG enrichment bubble graph, red boxed options are key signaling pathways. (**D**) Heat map.

**Figure 11:**
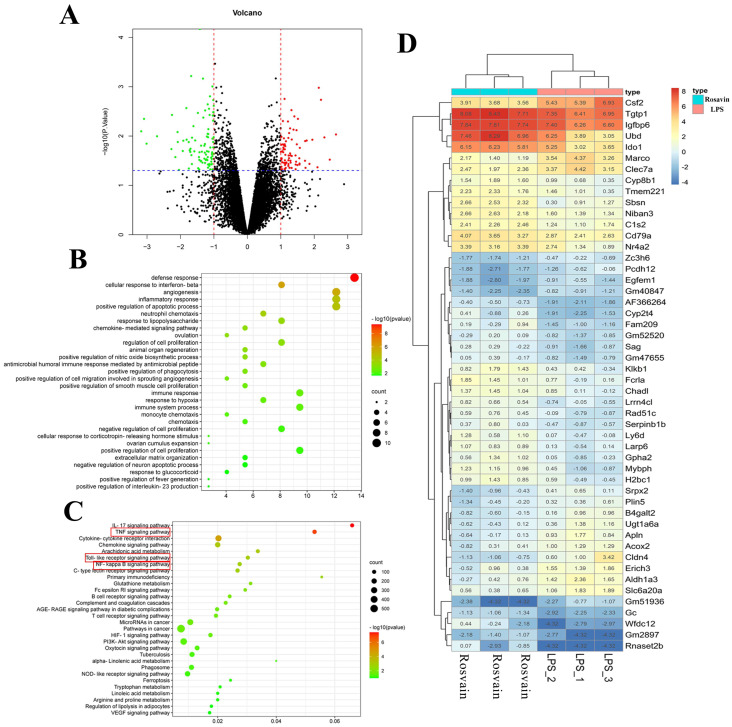
Transcriptomics analysis results of the LPS group and rosavin high dose group. (**A**) Volcano graph. Black dots represent genes, red dots represent up-regulated genes, green dots represent down-regulated genes, and the red line represents the screening screening condition of |log_2_FC| > 1, and the blue line represents the screening condition of *p* < 0.05. (**B**) GO biological process enrichment graph. (**C**) KEGG enrichment bubble graph, red boxed options are key signaling pathways. (**D**) Heat map.

RNA-seq analysis was performed in the control, LPS, and high-dose rosavin groups to better understand the mechanism of the protective effect of rosavin in mice with ALI. The findings revealed that 10,871 genes were variably expressed in the LPS group, compared with the control group, with |log_2_FC| > 1 and, *p* < 0.05 defined as differential genes. The total number of differentially expressed genes was 3085; 1427 genes were significantly up-regulated (*p* < 0.05), whereas 1658 genes were significantly down-regulated (*p* < 0.05). The resulting differential genes were closely associated with a variety of biological processes, including inflammatory, immune, and viral responses. There were 77 genes with changes in expression after rosavin treatment, compared with the LPS group, and 47 genes were significantly over-regulated, while 30 genes were significantly down-regulated. These differentially expressed genes were closely related to biological processes such as inflammatory response, and the regulation of the apoptotic process. According to GO functional enrichment analysis, the ability of rosavin to alleviate lipopolysaccharide-induced ALI may be linked to the involvement of inflammatory, immune, and apoptotic pathways. Furthermore, KEGG enrichment profiling of the resulting differential genes showed that they were closely associated with the Toll-like receptor signaling pathway, NF-κB signaling pathway, MAPK signaling pathway, and the apoptotic signaling pathway, implying that rosavin may affect these pathways and reduce ALI (Figure 10 and Figure 11).

#### 2.3.4. Rosavin Inhibits LPS-Induced Stimulation of the TLR-4/NF-κB Signaling Pathway in Mice

**Figure 12:**
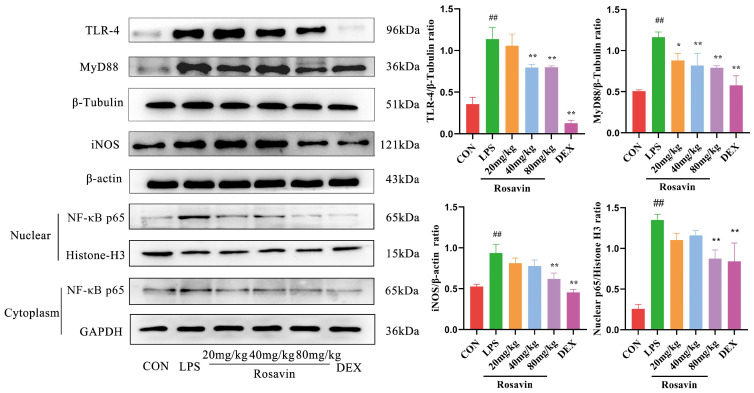
Effect of rosavin on TLR-4, MyD88, NF-κB p65, and iNOS protein expression in lung tissue. Data are shown as the mean ± standard deviation (*n* = 3); ^##^ *p* < 0.01 vs. control group; * *p* < 0.05, ** *p* < 0.01 vs. LPS group.

TLR-4, MyD88, and nuclear NF-κB p65 protein levels were increased in the LPS group (*p* < 0.01), while those in the rosavin-treated group were normalized (*p* < 0.05). Furthermore, the rosavin-treated group inhibited the increased NF-κB production of the downstream protein iNOS. These findings indicate that rosavin prevents LPS-induced lung TLR-4/NF-κB activation (Figure 12).

#### 2.3.5. Rosavin Inhibits LPS-Induced Activation of the MAPK Signaling Pathway in Mice

**Figure 13:**
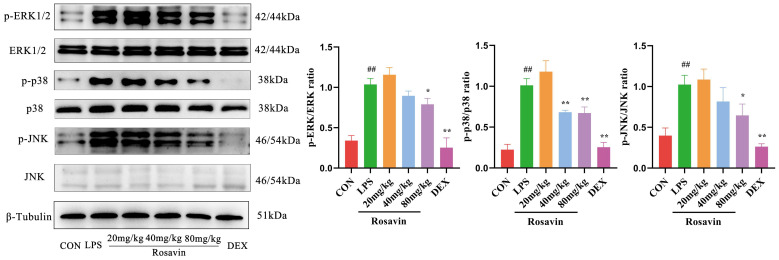
Effect of rosavin on p-ERK, p-p38, and p-JNK protein expression in lung tissue. Data are shown as the mean ± standard deviation (*n* = 3); ^##^ *p* < 0.01 vs. control group; * *p* < 0.05, ** *p* < 0.01 vs. LPS group.

We measured the expression of three MAPK proteins (ERK1/2, JNK, p38, and their phosphorylated versions) in mice. Lipopolysaccharide substantially enhanced MAPK phosphorylation compared with the control group (*p <* 0.01). Meanwhile, the phosphorylation of ERK1/2, JNK, and p38 was substantially lower in the rosavin-treated group (*p*< 0.05) (Figure 13).

#### 2.3.6. Rosavin Suppresses LPS-Induced Apoptosis in Mice

**Figure 14:**
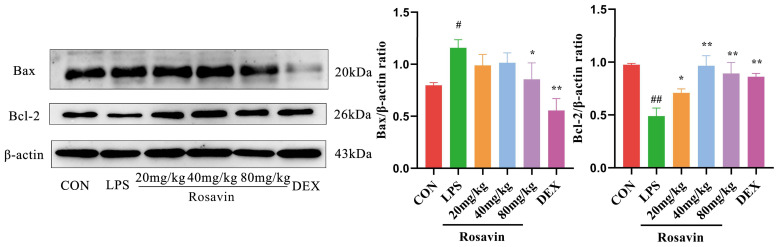
Effect of rosavin on Bax and Bcl-2 protein expression in lung tissue. Data are shown as the mean ± standard deviation (*n* = 3); ^#^ *p* < 0.05, ^##^ *p* < 0.01 vs. control group; * *p* < 0.05, ** *p* < 0.01 vs. LPS group.

Lipopolysaccharide treatment increased the expression of the pro-apoptotic protein Bax and decreased the expression of the anti-apoptotic protein Bcl-2, compared with the control group (*p* < 0.01). Lipopolysaccharide-induced apoptosis was greatly attenuated in the rosavin-treated group (*p* < 0.05) (Figure 14).

## 3. Discussion

ALI is a devastating respiratory disease characterized by uncontrolled inflammatory responses [29,30]. LPS is an important factor in triggering pneumonia and has been widely studied for its potential to cause endothelial barrier dysfunction; it can effectively activate immune cells via TLR-4 and induce a variety of pro-inflammatory mediators and cytokines through different signaling pathways [6,31]. The inflammation mediation is closely related to the TLR-4/MyD88/MAPK signaling pathway [32,33]. New anti-inflammatory drugs are being discovered based on the inhibition of the TLR-4/MyD88/MAPK signaling pathway. Rosavin is a phenylpropanoid and its glucoside is isolated from *Rhodiola rosea* L; it possesses a certain degree of anti-inflammatory activity [34]. In order to investigate the potential targets of rosavin’s protective effects against lung injury, a network pharmacological analysis was first performed, suggesting that the apoptosis and MAPK signaling pathways may be critical. Meanwhile, this study used LPS to construct an inflammation model in RAW264.7 and assessed the in vitro anti-inflammatory activity of rosavin. Lipopolysaccharide elevated ROS levels in RAW264.7 cells and mediated the elevation of downstream inflammatory factor NO levels through the activation of the TLR-4/NF-κB signaling pathway, whereas rosavin inhibited the activation of TLR-4//Myd88/NF-κB and reduced the activity of cyclooxygenase to contribute to anti-inflammatory progression.

Lung inflammation is a common pathophysiological feature of many respiratory diseases, including ALI [35]. IL-6 and TNF-α are the most potent inflammatory factors that can trigger, catalyze, and amplify the systemic inflammatory response [36,37]. Pro-inflammatory mediators such as macrophage inflammatory protein-3α (MIP-3α) and macrophage chemotactic protein (MCP-1) can cause PMN to adhere and cross the endothelial barrier to reach the site of inflammation, triggering an inflammatory storm [38]. CCXL2 is a well-known CC family chemokine with chemotactic activity against monocytes and basophils and is one of the key chemokines regulating monocyte/macrophage migration and infiltration [39]. These inflammatory cytokines are important and cause lung inflammation; therefore, the inhibition of the release of cells and inflammatory cytokines is an important measure to block the exacerbation of lung injury. This study used LPS to stimulate A549 cells to construct an in vitro model of lung injury; supernatant inflammatory factor assays showed that the levels of inflammatory factors in the LPS group were correspondingly increased, and proteins, such as MCP-1, CXCL-2, MIP-3α were obviously increased. Rosavin significantly reduced the level of inflammatory factors and the expression of chemokines. During the entire process of ALI, the basement membrane and alveolar capillary barrier are damaged and the permeability of alveolar capillaries is increased, which ultimately exacerbates the entry of inflammatory cells into the interstitial and alveolar cavities. No single marker or parameter has sufficient sensitivity and specificity to recognize the occurrence of all forms and severities of ALI. The currently accepted endpoint criteria fall into three categories: histologic evidence, alteration of the alveolar capillary barrier, and presence of inflammatory response and physiologic dysfunction, with histologic evidence demonstrated by alveolar or interstitial neutrophil. The histologic evidence of an inflammatory response is evidenced by the increased accumulation of neutrophils, increased extravascular content of the lungs, alterations in the alveolar capillary barrier, as evidenced by increased extravascular content of the lungs, increased total protein concentration in the bronchoalveolar fluid (BALF), and an increased wet/dry weight ratio of the lungs; the inflammatory response is evidenced by an increase in medullaryperoxidase (MPO) activity or protein concentration, an increase in the absolute number of neutrophils in the BALF, and increased concentration of pro-inflammatory cytokines in the lung tissues or in the BALF. The measurement of the above indicators is critical in order to determine whether ALI has occurred [14]. In this study, we found that after rosavin preventive treatment, the histopathological staining of the lungs demonstrated a remission of the inflammatory infiltrate, a significant reduction in the output of MPO, an improvement in the degree of edema of the lung tissues, and the proportion of inflammatory cells in the BALF was reduced, but the exact mechanism is still not clear enough. We used transcriptomics analysis to determine that the TLR-4 signaling pathway has an important molecular basis with rosavin-mediated inflammation. TLR-4 binding to LPS activates multiple signaling pathways [40,41]: MyD88 activates IκB kinase (IKK) through a signaling cascade, followed by the phosphorylation of the NF-κB inhibitor (IκB), the shedding of IκB from NF-κB, and the activation of NF-κB from its inhibitory state, which rapidly trans-locates to the nucleus to bind to the target protein and rapidly up-regulates the expression of pro-inflammatory cytokines such as IL-1β, TNF-α, IL-6 and IL-8 [42,43]. Jinping Liang [44] established a mouse model of ALI by treating mice with LPS via a non-exposed tracheal drip. The expression of TLR-4/MyD88/NF-κB proteins in mouse lung tissues was down-regulated after treating LPS-induced mice with rosavin [45]. This study found rosavin reduces the expression of inflammatory factors in the lungs with ALI by inhibiting the TLR-4/NF-κB signaling pathway, which attenuated the ALI inflammatory response and ameliorated lung injury.

MAPK signaling plays a critical role in various cellular processes such as apoptosis, survival, growth, and differentiation [46]. The MAPK signaling pathway is a downstream signaling cascade of the TLR-4-mediated inflammatory pathway and its major associated effector proteins include ERK, p38, and JNK [47]. MAPK is predominantly found in the cytoplasm of quiescent cells under normal conditions [48]. The MAPK pathway is the central signal transduction pathway and can be activated by a variety of stimuli, such as cytokines, radiation, and osmotic stress [49,50]. Activated MAPK receives signals converted and delivered by membrane receptors and carries them to the nucleus, where they play key roles in the production of inflammatory cytokines and other biological functions [18,51]. In contrast, JNK and p38 MAPK signaling is primarily activated by pro-inflammatory mediators and stress [52]. Similarly, our study showed that rosavin significantly inhibited the phosphorylation of ERK, JNK, and p38 in lung tissues, demonstrating that rosavin blocked the cascade of MAPK responses, mediating the synthesis and release of inflammatory factors and modulating neutrophil aggregation and migration to ameliorate pneumonitis in *Mycoplasma pneumoniae* infection. Stress-activated p38/JNK MAPK plays a key role in balancing cell survival and death in response to extracellular and intracellular stress. Bcl-2 is an anti-apoptotic protein that stabilizes the mitochondrial membrane, whereas Bax is a pro-apoptotic protein that increases the permeability of the mitochondrial membrane [53,54]. p38 and JNK induces the release of Bax and its translocation to the mitochondria, which promotes apoptosis [54]. Rosavin can downregulate Bax and upregulate Bcl-2 protein expression. Rosavin effectively attenuates apoptosis and ameliorates ALI.

**Figure 15:**
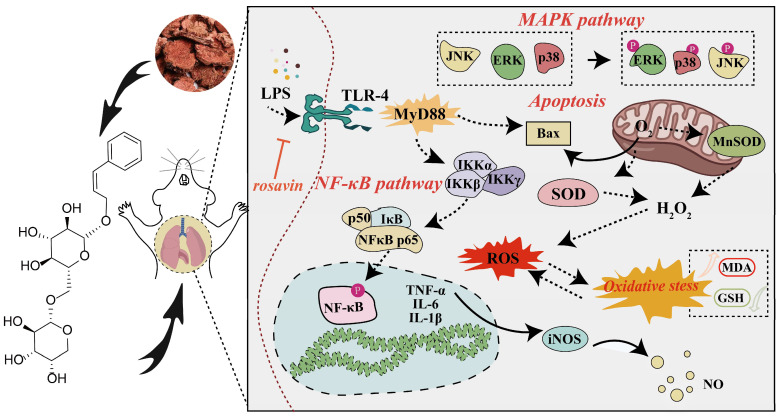
Schematic diagram of the molecular mechanism of rosavin alleviated LPS-induced ALI.

Guidelines for the treatment of ALI/ARDS-mediated inflammation suggest that the mainstay of treatment is anti-inflammatory therapy with antibiotics and glucocorticoids [55]. In this study, compared with dexamethasone, we found the effect of rosavin on inhibiting TLR-4/NF-κB/MAPK and apoptosis signaling pathways in ALI inflammation as shown in Figure 15. It provides a new perspective for the study of the mechanism of action of rosavin and lays a mechanistic foundation for clinical applications in the therapeutic intervention of acute lung injury. This research is crucial to our understanding of the pathophysiology of ALI and the development of new therapeutic strategies for human ALI/ARDS. How to apply these applications with findings from animal models to humans is even more worthy of consideration along with long-term experimental or potential toxicology experimental data and specific molecular mechanism research. We will further improve the above key issues in the subsequent experiments.

## 4. Materials and Methods

### 4.1. Chemicals and Reagents

Rosavin is a laboratory isolate with a purity of over 98% (Supplementary Part SI Figures S1–S3). Lipopolysaccharide (O55: B5 0000114326) was purchased from Sigma Aldrich, St. Louis, MO, USA. Mouse IL-6 (A20610435), IL-1β (A201B10612), and TNF-α enzyme linked immunosorbent assay (ELISA) kits (A28210444) were purchased from LianKe Biotechnology Co., Ltd., Hangzhou, China. Total Superoxide Dismutase (T-SOD; A001-1-2), glutataione (GSH; A006-2-1), malondialdehyde (MDA; A003-1-2), and myeloperoxidase (MPO) assay kits (A044-1-1) were purchased from Nanjing Jiancheng Biotechnology Co., Ltd., Nanjing, China. ROS detection kit (S0033S) was purchased from Beyotime Biotechnology Co., Ltd., Haimen, China. Mouse Anti-β-Tubulin (TUBB4) antibody (TA-10), mouse Anti-β-actin antibody (TA-09), mouse anti-GAPDH antibody (TA-08), goat anti-mouse antibody (127655), and goat anti-rabbit antibody (129256) were purchased from Beijing Zhongshan Jinqiao Biotechnology Co., Ltd., Beijing, China. ERK (BM4326), MyD88 (BA2321), p38 (BM4142), Bax (A00183), Bcl-2 (BA0412), iNOS (BA0360), COX-2 (BA3708), and CXCL-2 (PB0900) antibody were purchased from Boster Biotechnology Co., Ltd., Wuhan, China. TLR-4 (19811-1-AP), JNK (66210-1-1G), TNF-α (60291-1-1g), and Histone-H3 antibody (17168-1-AP) were purchased from Proteintech Biotechnology Co., Ltd., Wuhan, China. NF-κB p65 (8242S), phosphorylated JNK (4668S), phosphorylated ERK (143707), phosphorylated p38 (4631S) antibody were purchased from Cell Signaling Biotechnology Co., Ltd. MCP-1 (AB25124) and MIP-3α (AB9829) antibody were purchased from Abcam Biotechnology Co. Ltd., Cambridge, UK. Enhanced chemi-luminescence (ECL) Western blotting substrate (KF8005) was purchased from Affinity Biotechnology Co., Ltd., Cincinnati, OH, USA.

### 4.2. Network Pharmacology Analysis

Rosavin structural information was used in the Swiss Target Prediction database (http://www.swisstargetprediction.c) to identify targets. GeneCards (https://www.genecards.org/) and the OMIM database (https://www.omim.org/) were utilized to determine ALI relevant targets accessed on 1 June 2021. The intersecting targets of rosavin and ALI were analyzed using Venn analysis. With the qualification that the species was human, and the confidence score was greater than 0.9, the protein-protein interaction (PPI) network was analyzed by using the STRING database (https://cn.string-db.org/). The final key targets were analyzed using the DAVID (https://david.ncifcrf.gov/) and KOBAS (http://kobas.cbi.pku.edu.cn/) databases for Gene Ontology (GO) and Kyoto Encyclopedia of Genes and Genomes (KEGG) enrichment annotation to predict the possible targets of rosavin accessed on 15 June 2021.

### 4.3. In Vitro Study

#### 4.3.1. Cell Culture and Treatment

Mouse macrophage cell RAW264.7 was purchased from the Cell Bank of the Chinese Academy of Sciences and cultured in DMEM high glucose growth medium (BI, Akko, Israel), which was supplemented with 10% FBS (BI, Akko, Israel), 1% penicillin (100 U/mL), and streptomycin (100 μg/mL).

Human alveolar basal epithelial cell A549 was purchased from the Cell Bank of the Chinese Academy of Sciences and cultured in DMEM/F12 growth medium (BI, Akko, Israel), which was supplemented with 10% FBS (BI, Akko, Israel), 1% penicillin (100 U/mL), and 100 μg/mL streptomycin.

RAW264.7 cells and A549 cells were treated with 0, 16, 32, 64 μM rosavin and 1 μg/mL LPS for 24 h [17,56], then cells or supernatants were collected for subsequent processing [57].

#### 4.3.2. MTT Assay

RAW264.7 and A549 cells were seeded in 96-well plates (1 × 10^4^ cells/well) for 24 h, then treated with different concentrations of rosavin (0, 2, 4, 8, 16, 32, 64, 128 μM) for 24 h. Further, 10 µL of MTT (5 mg/mL) was added to each well and incubated for 4 h at 37 °C. Next, 100 µL of DMSO was added to each well to dissolve the formazan. Absorbance was measured at 490 nm using a microplate reader (3001; Thermo Fisher Scientific, Waltham, MA, USA).

#### 4.3.3. Nitric Oxide Detection

An NO assay kit (S0021, Beyotime) was used to detect the levels of nitric oxide. RAW264.7 cells were seeded in 24-well plates (2 × 10^5^ cells/well) for 24 h and treated with 64 µM rosavin and 1 μg/mL LPS for 24 h. The supernatant was collected and added to Griess Reagent I and Griess Reagent II sequentially, then incubated at 37 °C for 30 min, the absorbance was measured at 540 nm using a microplate reader.

#### 4.3.4. Dectection of ROS

RAW264.7 cells were seeded in a 24-well plate (2 × 10^5^ cells/well) for 24 h and treated with 64 µM rosavin and 1 μg/mL LPS for 24 h. The treated cells were collected, washed three times with PBS, and stained with 500 μL PBS containing 10 μM dichlorodihydrofluorescein diacetate (DCFH-DA) for 60 min in the dark. The cells were washed three times with PBS to remove the DCFH-DA and analyzed using a microplate reader or fluorescence microscope (Axio Imager A2; ZEISS, Macquarie Park, NSW, Australian).

#### 4.3.5. Cytokines’ Assay

A549 cells were seeded in 24-well plates (2 × 10^5^ cells/well) for 24 h and treated with 0, 16, 32, 64 μM rosavin and 1 μg/mL LPS for 24 h. The supernatant was collected, and the concentration of inflammatory factors (IL-6, TNF-α) in the supernatants was determined.

#### 4.3.6. Immunofluorescence

RAW264.7 cells were seeded on 24-well plates (2 × 10^5^ cells/well) for 24 h, and treated with 0, 16, 32, 64 μM rosavin and 1 μg/mL LPS for 24 h. The cells were fixed with 4% paraformaldehyde for 20 min at 37 °C, the membranes were broken with 0.5% Triton X-100 for 20 min, washed three times with PBS, and blocked with 1% BSA for 60 min at 37 °C. The cells were incubated with p65 antibody at 4 °C overnight, washed three times with PBS, and subsequently incubated with fluorescent secondary antibody for 90 min at 37 °C. The nuclei of the cells were stained with 4′, 6-diamidino-2-phenylindole (DAPI), and the slices were sealed with anti-fluorescence quenching sealer and observed under a fluorescence microscope (Axio Imager A2; ZEISS, Macquarie Park, NSW, Australian).

### 4.4. In Vivo Study

#### 4.4.1. Animal Groups and Treatment

**Figure 16:**
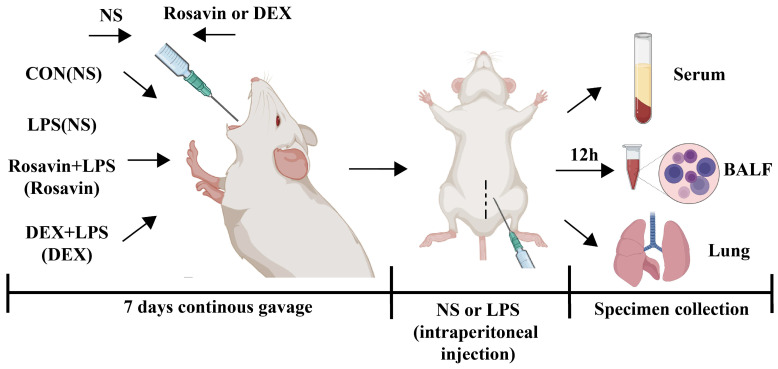
Laboratory flow diagram.

Six-to-eight-week-old SPF-grade male Balb/c mice (18 ± 22 g) were obtained from the Animal Experimentation Center of Xinjiang Medical University (No. SCXK (Xin) 2018-0001). Animal care and experimental procedures were approved by the Experimental Animal Ethics Committee of the First Hospital of Shihezi University School of Medicine (No. A2023-149-01, Supplementary Part SII). The mice were divided into six groups (n = 6 per group): (1) control group (CON); (2) LPS-challenged mice (LPS) (3) LPS-challenged mice were gavaged with 20 mg/kg rosavin; (4) LPS challenged mice were gavaged with 40 mg/kg rosavin; (5) LPS challenged mice were gavaged with 80 mg/kg rosavin; (6) LPS challenged mice were gavaged with 1 mg/kg dexamethasone (DEX). The dosage of rosavin is referenced in the following literature [27,28]. Mice in each dose group and the dexamethasone group were gavaged with the corresponding drugs once a day for seven consecutive days, and control mice were gavaged with an equal amount of solvent once a day for seven consecutive days. One hour after administration on the seventh day, LPS (5 mg/kg) solution was injected intraperitoneally into all groups except the control group, and the alveolar lavage fluid and lung tissues were collected after 12 h [18,34]. The flowchart of the animal experiment is shown in Figure 16.

#### 4.4.2. BALF Preparation and Examination

After euthanasia, the airways were exposed and the unwanted connective tissue on the bronchial tubes was peeled off. An 18G cannula needle was inserted into the trachea, and bronchoalveolar lavage was performed using 1.0 mL of pre-cooled PBS. The centrifuged precipitate was collected, re-suspended in PBS, and subjected to Wright–Giemsa staining.

#### 4.4.3. Lung Wet/Dry Weight Proportion Analysis

Lung tissue was accurately weighed using an analytical balance to determine wet weight (W). The tissues were dried at 80 °C for 48 h. The weight (D) was recorded, and the wet weight/dry weight (W/D) ratio was computed.

#### 4.4.4. Histopathological Analysis

Mouse lung tissues were immobilized in 4% paraformaldehyde, entrenched in preheated paraffin wax and sectioned after the tissue wax block had solidified to a thickness of 5 µm (LEICA RM2245; Arbor Electronics, Lake Kiowa, TX, USA). The samples were dewaxed, and the sections were stained by hematoxylin and eosin (H&E) staining. Pathological changes in the lung tissue were observed (histopathological assessment was double-blinded and carried out by two pathologists), including affluent alveolar congestion, pulmonary edema, inflammatory cell infiltration, and mesenchymal hyperplasia, to determine lung injury.

#### 4.4.5. Enzyme-Linked Immunosorbent (ELISA) Assay

Serum levels of inflammatory cytokines (IL-6, IL-1β, and TNF-α) were quantified according to the manufacturer’s instructions.

#### 4.4.6. Determination of Myeloperoxidase (MPO), Superoxide Dismutase (SOD), Malondialdehyde (MDA) and Glutathione (GSH) in Lung Tissue

Tissue homogenates (10%) were prepared, and MPO, T-SOD activity, MDA and GSH levels were measured in the lung tissue according to the specific requirements stated in the instructions.

#### 4.4.7. Western Blotting

Total protein was extracted using high-efficiency tissue RIPA solution, and separated by 7.5–15% sodium dodecyl sulfate polyacrylamide gel electrophoresis (SDS-PAGE) to separate the proteins. The electrophoresis was stopped when bromophenol blue was separated from the bottom of the gel. A suitable loading control was selected according to the molecular weight of the target protein and the gel was cut. The corresponding size of the polyvinylidene difluoride (PVDF) membrane was attached for the electro-transfer of the proteins to the membrane. Membranes were blocked in 5% bovine serum protein at room temperature, incubated with the corresponding primary antibodies (dilution ratio: β-actin: 1:1000, β-Tubulin: 1:1000, GAPDH: 1:1000, NF-κB p65: 1:500, TLR-4: 1:500, MyD88: 1:500, Bax: 1:500, Bcl-2: 1:500, iNOS: 1:500, p38: 1:500, p-p38: 1:500, ERK: 1:500, p-ERK: 1:500, JNK: 1:500, p-JNK: 1:500, MCP-1: 1:1000, MIP-3α: 1:1000, COX-2: 1:500, TNF-α: 1:500, CXCL-2: 1:1000), and incubated with goat anti-mouse/rabbit antibodies (dilution ratio: 1:10,000) on a shaker for 1.5 h at room temperature. Images were captured using a gel imaging system and analyzed using Image-Pro 6.0 software.

#### 4.4.8. Nucleocytoplasmic Separation

Nuclear and cytoplasmic proteins were isolated from the lung tissue using the Nuclear Protein Extraction Kit, according to the manufacturer’s instructions (R0050; Solarbio, Beijing, China). The expression levels of p65, GAPDH, and Histone H3 were evaluated by Western blot analysis.

#### 4.4.9. Transcriptome Sequencing

RNA was isolated from the lung tissues of control, LPS, and rosavin (80 mg/kg) mice using TRIzol reagent. An Agilent 2100 Bioanalyzer was used to detect the range of the inserted fragments in the library. The ABI Step One Plus Real-Time PCR System (TaqMan Probe, v7500) was used to quantify library concentrations, and Illumina HiSeq^TM^ 2500 was used for library sequencing. The screened genomic data were compared to the reference database using HISAT2. Transcriptome data were collected after comparison, and differential expression analysis was performed using DESeq2 (v1.4.5) (screening criteria were more than 2-fold change, *p* < 0.05). Gene Ontology functional enrichment and KEGG pathway enrichment analysis were conducted using R software(4.2.1).

### 4.5. Statistical Analysis

GraphPad Prism 8 (San Diego, CA, USA) was used for the statistical examination of the data, which was stated as the mean ± standard deviation. The data were screened for significant differences using one-way analysis of variance (ANOVA). A statistically significant difference was identified as *p* < 0.05. A significant difference in the data was interpreted as *p* < 0.01.

## 5. Conclusions

In our study, we found that rosavin was able to eliminate acute lung injury by inhibiting the activation of the TLR-4/NF-κB/MAPK signaling pathway and apoptosis, which not only provides theoretical ideas for elucidating the possible molecular mechanisms of rosavin but also provides a rationale for the potential clinical application of rosavin in the future treatment of ALI.

## Data Availability

Data is contained within the article and supplementary material.

## Supplementary Materials

The following supporting information can be downloaded at: https://www.mdpi.com/article/10.3390/ijms25031875/s1.

## References

[1] Ding Z., Zhong R., Yang Y., Xia T., Wang W., Wang Y., Xing N., Luo Y., Li S., Shang L. (2020). Systems pharmacology reveals the mechanism of activity of Ge-Gen-Qin-Lian decoction against LPS-induced acute lung injury: A novel strategy for exploring active components and effective mechanism of TCM formulae. Pharmacol. Res..

[2] Huang C.-Y., Deng J.-S., Huang W.-C., Jiang W.-P., Huang G.-J. (2020). Attenuation of Lipopolysaccharide-Induced Acute Lung Injury by Hispolon in Mice, Through Regulating the TLR4/PI3K/Akt/mTOR and Keap1/Nrf2/HO-1 Pathways, and Suppressing Oxidative Stress-Mediated ER Stress-Induced Apoptosis and Autophagy. Nutrients.

[3] Qian Y., Wang Z., Lin H., Lei T., Zhou Z., Huang W., Wu X., Zuo L., Wu J., Liu Y. (2022). TRIM47 is a novel endothelial activation factor that aggravates lipopolysaccharide-induced acute lung injury in mice via K63-linked ubiquitination of TRAF2. Signal Transduct. Target. Ther..

[4] Zhao R., Wang B., Wang D., Wu B., Ji P., Tan D. (2021). Oxyberberine Prevented Lipopolysaccharide-Induced Acute Lung Injury through Inhibition of Mitophagy. Oxidative Med. Cell. Longev..

[5] Long M.E., Mallampalli R.K., Horowitz J.C. (2022). Pathogenesis of pneumonia and acute lung injury. Clin. Sci..

[6] Zhang J., Zhang M., Zhang W.-H., Zhu Q.-M., Ning J., Huo X.-K., Xiao H.-T., Sun C.-P. (2022). Total terpenoids of Inula japonica activated the Nrf2 receptor to alleviate the inflammation and oxidative stress in LPS-induced acute lung injury. Phytomedicine.

[7] Liu Y., Zhou J., Luo Y., Li J., Shang L., Zhou F., Yang S. (2021). Honokiol alleviates LPS-induced acute lung injury by inhibiting NLRP3 inflammasome-mediated pyroptosis via Nrf2 activation in vitro and in vivo. Chin. Med..

[8] Yang C., Song C., Liu Y., Qu J., Li H., Xiao W., Kong L., Ge H., Sun Y., Lv W. (2021). Re-Du-Ning injection ameliorates LPS-induced lung injury through inhibiting neutrophil extracellular traps formation. Phytomedicine.

[9] Wang Y., Wang X., Li Y., Xue Z., Shao R., Li L., Zhu Y., Zhang H., Yang J. (2022). Xuanfei Baidu Decoction reduces acute lung injury by regulating infiltration of neutrophils and macrophages via PD-1/IL17A pathway. Pharmacol. Res..

[10] Zhang J., Zhang M., Huo X.-K., Ning J., Yu Z.-L., Morisseau C., Sun C.-P., Hammock B.D., Ma X.-C. (2023). Macrophage Inactivation by Small Molecule Wedelolactone via Targeting sEH for the Treatment of LPS-Induced Acute Lung Injury. ACS Cent. Sci..

[11] Liu C., Yin Z., Feng T., Zhang M., Zhou Z., Zhou Y. (2021). An integrated network pharmacology and RNA-Seq approach for exploring the preventive effect of Lonicerae japonicae flos on LPS-induced acute lung injury. J. Ethnopharmacol..

[12] Zhang H., Lang W., Wang S., Li B., Li G., Shi Q. (2020). Echinacea polysaccharide alleviates LPS-induced lung injury via inhibiting inflammation, apoptosis and activation of the TLR4/NF-κB signal pathway. Int. Immunopharmacol..

[13] Zhang J., Guo Y., Mak M., Tao Z. (2024). Translational medicine for acute lung injury. J. Transl. Med..

[14] Matute-Bello G., Downey G., Moore B.B., Groshong S.D., Matthay M.A., Slutsky A.S., Kuebler W.M. (2011). An Official American Thoracic Society Workshop Report: Features and Measurements of Experimental Acute Lung Injury in Animals. Am. J. Respir. Cell Mol. Biol..

[15] Wang Y.-W., Wu Y.-H., Zhang J.-Z., Tang J.-H., Fan R.-P., Li F., Yu B.-Y., Kou J.-P., Zhang Y.-Y. (2020). Ruscogenin attenuates particulate matter-induced acute lung injury in mice via protecting pulmonary endothelial barrier and inhibiting TLR4 signaling pathway. Acta Pharmacol. Sin..

[16] Miao J., Shen J., Yan C., Ren J., Liu H., Qiao Y., Li Q. (2022). The protective effects of Mai-Luo-Ning injection against LPS-induced acute lung injury via the TLR4/NF-κB signalling pathway. Phytomedicine.

[17] Cai J., Wang Y.-l., Sheng X.-d., Zhang L., Lv X. (2022). Shufeng Jiedu capsule inhibits inflammation and apoptosis by activating A2AAR and inhibiting NF-κB to alleviate LPS-induced ALI. J. Ethnopharmacol..

[18] Hong H., Lou S., Zheng F., Gao H., Wang N., Tian S., Huang G., Zhao H. (2022). Hydnocarpin D attenuates lipopolysaccharide-induced acute lung injury via MAPK/NF-κB and Keap1/Nrf2/HO-1 pathway. Phytomedicine.

[19] Zhu W., Wang M., Jin L., Yang B., Bai B., Mutsinze R.N., Zuo W., Chattipakorn N., Huh J.Y., Liang G. (2022). Licochalcone A protects against LPS-induced inflammation and acute lung injury by directly binding with myeloid differentiation factor 2 (MD2). Br. J. Pharmacol..

[20] Shi K., Xiao Y., Dong Y., Wang D., Xie Y., Tu J., Xu K., Zhou Z., Cao G., Liu Y. (2022). Protective Effects of Atractylodis lancea Rhizoma on Lipopolysaccharide-Induced Acute Lung Injury via TLR4/NF-κB and Keap1/Nrf2 Signaling Pathways In Vitro and In Vivo. Int. J. Mol. Sci..

[21] He Y.-Q., Zhou C.-C., Yu L.-Y., Wang L., Deng J.-L., Tao Y.-L., Zhang F., Chen W.-S. (2021). Natural product derived phytochemicals in managing acute lung injury by multiple mechanisms. Pharmacol. Res..

[22] Li C., Huang Y., Yao X., Hu B., Wu S., Chen G., Lv X., Tian F. (2016). Lugrandoside attenuates LPS-induced acute respiratory distress syndrome by anti-inflammation and anti-apoptosis in mice. Am. J. Transl. Res..

[23] Qi Z., Qi S., Ling L., Lv J., Feng Z. (2016). Salidroside attenuates inflammatory response via suppressing JAK2-STAT3 pathway activation and preventing STAT3 transfer into nucleus. Int. Immunopharmacol..

[24] Zhang H., Dong W., Li S., Zhang Y., Lv Z., Yang L., Jiang L., Wu T., Wang Y. (2021). Salidroside protects against ventilation-induced lung injury by inhibiting the expression of matrix metalloproteinase-9. Pharm. Biol..

[25] Wang Y., Xu Y., Zhang P., Ruan W., Zhang L., Yuan S., Pang T., Jia A.-Q. (2018). Smiglaside A ameliorates LPS-induced acute lung injury by modulating macrophage polarization via AMPK-PPARγ pathway. Biochem. Pharmacol..

[26] Gao T., Li J., Shi L., Hu B. (2023). Rosavin inhibits neutrophil extracellular traps formation to ameliorate sepsis-induced lung injury by regulating the MAPK pathway. Allergol. Et Immunopathol..

[27] Wang Y., Zhao S., Jia N., Shen Z., Huang D., Wang X., Wu Y., Pei C., Shi S., He Y. (2022). Pretreatment with rosavin attenuates PM2.5-induced lung injury in rats through antiferroptosis via PI3K/Akt/Nrf2 signaling pathway. Phytother. Res..

[28] Xin X., Yao D., Zhang K., Han S., Liu D., Wang H., Liu X., Li G., Huang J., Wang J. (2019). Protective effects of Rosavin on bleomycin-induced pulmonary fibrosis via suppressing fibrotic and inflammatory signaling pathways in mice. Biomed. Pharmacother..

[29] Rao Z., Li X., Zhang X., Zeng J., Wang B., Yang R., Zeng N. (2022). Fengreqing Oral Liquid Exerts Anti-Inflammatory Effects by Promoting Apoptosis and Inhibiting PI3K/AKT and NF-κB Signaling Pathways. Front. Pharmacol..

[30] Yu Y.-Y., Li X.-Q., Hu W.-P., Cu S.-C., Dai J.-J., Gao Y.-N., Zhang Y.-T., Bai X.-Y., Shi D.-Y. (2022). Self-developed NF-κB inhibitor 270 protects against LPS-induced acute kidney injury and lung injury through improving inflammation. Biomed. Pharmacother..

[31] Wang J., Yang H., Zheng D., Sun Y., An L., Li G., Zhao Z. (2023). Integrating network pharmacology and pharmacological evaluation to reveal the therapeutic effects and potential mechanism of S-allylmercapto-N-acetylcysteine on acute respiratory distress syndrome. Int. Immunopharmacol..

[32] Zhu W., Luo W., Han J., Zhang Q., Ji L., Samorodov A.V., Pavlov V.N., Zhuang Z., Yang D., Yin L. (2023). Schisandrin B protects against LPS-induced inflammatory lung injury by targeting MyD88. Phytomedicine.

[33] Han S., Yuan R., Cui Y., He J., Wang Q.-Q., Zhuo Y., Yang S., Gao H. (2022). Hederasaponin C Alleviates Lipopolysaccharide-Induced Acute Lung Injury In Vivo and In Vitro Through the PIP2/NF-κB/NLRP3 Signaling Pathway. Front. Immunol..

[34] Jia X., Zhang K., Feng S., Li Y., Yao D., Liu Q., Liu D., Li X., Huang J., Wang H. (2023). Total glycosides of Rhodiola rosea L. attenuate LPS-induced acute lung injury by inhibiting TLR4/NF-κB pathway. Biomed. Pharmacother..

[35] Yang L., Chen H., Hu Q., Liu L., Yuan Y., Zhang C., Tang J., Shen X. (2022). Eupalinolide B attenuates lipopolysaccharide-induced acute lung injury through inhibition of NF-κB and MAPKs signaling by targeting TAK1 protein. Int. Immunopharmacol..

[36] Chen J., Huang Y., Bian X., He Y. (2022). Berberine Ameliorates Inflammation in Acute Lung Injury via NF-κB/Nlrp3 Signaling Pathway. Front. Nutr..

[37] Liu L., Chen X., Jiang Y., Yuan Y., Yang L., Hu Q., Tang J., Meng X., Xie C., Shen X. (2022). Brevilin A Ameliorates Acute Lung Injury and Inflammation Through Inhibition of NF-κB Signaling via Targeting IKKα/β. Front. Pharmacol..

[38] Liao X., Zhang W., Dai H., Jing R., Ye M., Ge W., Pei S., Pan L. (2021). Neutrophil-Derived IL-17 Promotes Ventilator-Induced Lung Injury via p38 MAPK/MCP-1 Pathway Activation. Front. Immunol..

[39] Kim S.-H., Hong J.-H., Yang W.-K., Geum J.-H., Kim H.-R., Choi S.-Y., Kang Y.-M., An H.-J., Lee Y.-C. (2020). Herbal Combinational Medication of Glycyrrhiza glabra, Agastache rugosa Containing Glycyrrhizic Acid, Tilianin Inhibits Neutrophilic Lung Inflammation by Affecting CXCL2, Interleukin-17/STAT3 Signal Pathways in a Murine Model of COPD. Nutrients.

[40] Yang J., Wang M., Xu Y., Liao J., Li X., Zhou Y., Dai J., Li X., Chen P., Chen G. (2023). Discovery of 4-oxo-N-phenyl-1,4-dihydroquinoline-3-carboxamide derivatives as novel anti-inflammatory agents for the treatment of acute lung injury and sepsis. Eur. J. Med. Chem..

[41] Lee H.L., Kim J.M., Go M.J., Kim T.Y., Joo S.G., Kim J.H., Lee H.S., Kim H.-J., Heo H.J. (2023). Protective Effect of Lonicera japonica on PM2.5-Induced Pulmonary Damage in BALB/c Mice via the TGF-β and NF-κB Pathway. Antioxidants.

[42] Liao J., Yang J., Li X., Hu C., Zhu W., Zhou Y., Zou Y., Guo M., Chen Z., Li X. (2023). Discovery of the Diphenyl 6-Oxo-1,6-dihydropyridazine-3-carboxylate/carboxamide Analogue J27 for the Treatment of Acute Lung Injury and Sepsis by Targeting JNK2 and Inhibiting the JNK2-NF-κB/MAPK Pathway. J. Med. Chem..

[43] Cui B., Liu Y., Chen J., Chen H., Feng Y., Zhang P. (2023). Small molecule inhibitor CRT0066101 inhibits cytokine storm syndrome in a mouse model of lung injury. Int. Immunopharmacol..

[44] Liang J., Liu J., Tang Y., Peng Q., Zhang L., Ma X., Xu N., Wei J., Han H. (2022). Sophoridine inhibits endotoxin-induced acute lung injury by enhancing autophagy of macrophage and reducing inflammation. J. Leukoc. Biol..

[45] Chen X., Zhao Y., Wang X., Lin Y., Zhao W., Wu D., Pan J., Luo W., Wang Y., Liang G. (2022). FAK mediates LPS-induced inflammatory lung injury through interacting TAK1 and activating TAK1-NFκB pathway. Cell Death Dis..

[46] Jangam A., Tirunavalli S.K., Adimoolam B.M., Kasireddy B., Patnaik S.S., Erukkambattu J., Thota J.R., Andugulapati S.B., Addlagatta A. (2023). Anti-inflammatory and antioxidant activities of Gymnema Sylvestre extract rescue acute respiratory distress syndrome in rats via modulating the NF-κB/MAPK pathway. Inflammopharmacology.

[47] Zhu X., Bai B., Ge X., Zheng B., Xiao Z., Tang Y., Fang L., Tang Y., Dai Y., Zhang B. (2023). Costunolide attenuates LPS-induced inflammation and lung injury through inhibiting IKK/NF-κB signaling. Naunyn-Schmiedeberg’s Arch. Pharmacol..

[48] Zhao L., Zhang Z., Li P., Gao Y., Shi Y. (2023). Bakuchiol regulates TLR4/MyD88/NF-κB and Keap1/Nrf2/HO-1 pathways to protect against LPS-induced acute lung injury in vitro and in vivo. Naunyn-Schmiedeberg’s Arch. Pharmacol..

[49] Li Y.-L., Qin S.-Y., Li Q., Song S.-J., Xiao W., Yao G.-D. (2023). Jinzhen Oral Liquid alleviates lipopolysaccharide-induced acute lung injury through modulating TLR4/MyD88/NF-κB pathway. Phytomedicine.

[50] Guo Y., Zhang H., Lv Z., Du Y., Li D., Fang H., You J., Yu L., Li R. (2023). Up-regulated CD38 by daphnetin alleviates lipopolysaccharide-induced lung injury via inhibiting MAPK/NF-κB/NLRP3 pathway. Cell Commun. Signal..

[51] Pei X., Zhang Z., Wang N., Huang G., Min X., Yang Y., Cao J. (2023). Onychiol B attenuates lipopolysaccharide-induced inflammation via MAPK/NF-κB pathways and acute lung injury in vivo. Bioorg. Chem..

[52] Cui Y.-R., Qu F., Zhong W.-J., Yang H.-H., Zeng J., Huang J.-H., Liu J., Zhang M.-Y., Zhou Y., Guan C.-X. (2022). Beneficial effects of aloperine on inflammation and oxidative stress by suppressing necroptosis in lipopolysaccharide-induced acute lung injury mouse model. Phytomedicine.

[53] Xu X.J., Zhang M.L., Hou Y.M., Zhang K., Yao D.H., Li G.Y., Kou W.B., Wang H.Y., Wang J.H. (2022). The Amomum tsao-ko Essential Oils Inhibited Inflammation and Apoptosis through p38/JNK MAPK Signaling Pathway and Alleviated Gentamicin-Induced Acute Kidney Injury. Molecules.

[54] Li W., Li D., Chen Y., Abudou H., Wang H., Cai J., Wang Y., Liu Z., Liu Y., Fan H. (2022). Classic Signaling Pathways in Alveolar Injury and Repair Involved in Sepsis-Induced ALI/ARDS: New Research Progress and Prospect. Dis. Markers.

[55] Bos L.D.J., Ware L.B. (2022). Acute respiratory distress syndrome: Causes, pathophysiology, and phenotypes. Lancet.

[56] Wang J., Ren C., Bi W., Batu W. (2023). Glycyrrhizin mitigates acute lung injury by inhibiting the NLRP3 inflammasome in vitro and in vivo. J. Ethnopharmacol..

[57] Xi X., Yao Y., Liu N., Li P. (2020). MiR-297 alleviates LPS-induced A549 cell and mice lung injury via targeting cyclin dependent kinase 8. Int. Immunopharmacol..

