# Supplementary material for: Rosavin Alleviates LPS-Induced Acute Lung Injure by Modulating the TLR-4/NF-κB/MAPK Singnaling Pathways
Source: Int J Mol Sci. 2024 Feb 3;25(3):1875. doi: 10.3390/ijms25031875 (PMC10856478; doi:10.3390/ijms25031875)
Supplement: Supplementary file 1 [file ijms-25-01875-s001.zip › Original Images for Blots Gels.pdf]

## The original Western blots uncropped image

**Figure1**.Panels A-F represent the manuscript entitled "Rosavin alleviates LPS-induced acute lung injure by modulating the TLR-4/NF-κB/MAPK singnaling pathways" in figure 3. The entire uncropped images of the original Western blots shown are repeated three times for each protein, as shown in Test 1, Test 2, and Test 3. A: TLR-4; B: MyD88; C: β-Tubulin; D: COX-2; E: TNF-α; F: β-actin. The order of samples was CON, LPS, rosavin (16 μM), rosavin (32 μM), rosavin (64 μM).

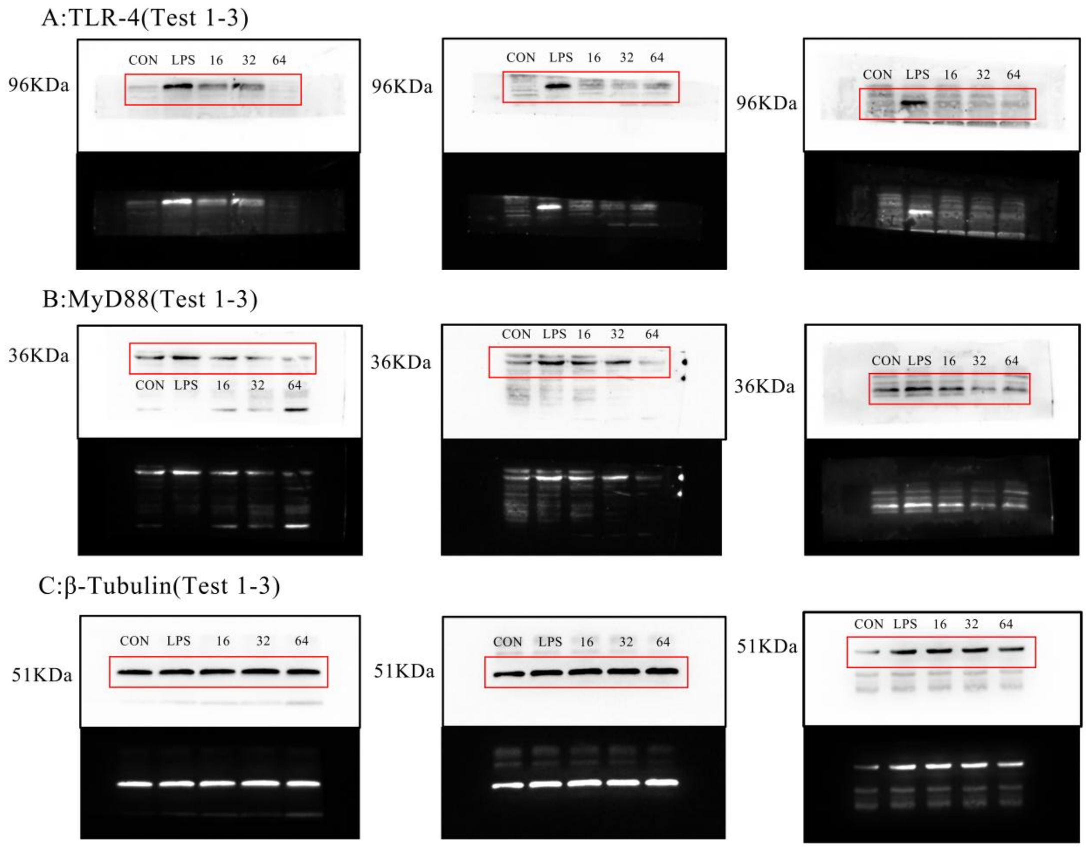

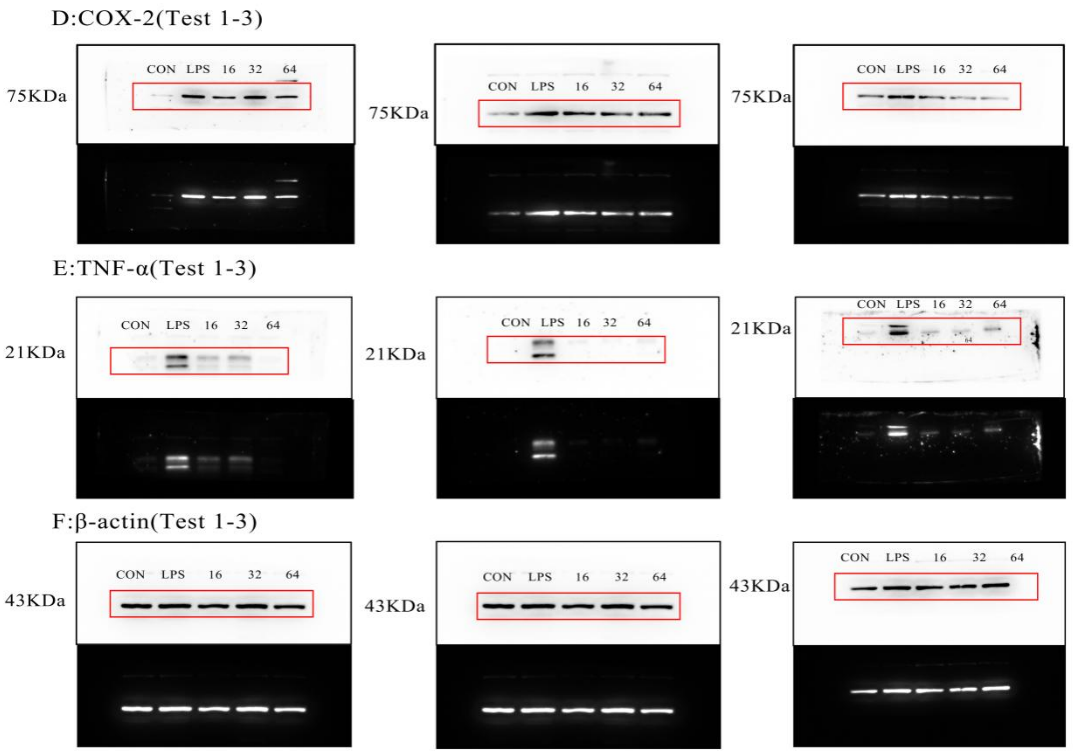


**Figure2**.Panels A-F represent the manuscript entitled "Rosavin alleviates LPS-induced acute lung injure by modulating the TLR-4/NF-κB/MAPK singnaling pathways" in figure 4. The entire uncropped images of the original Western blots shown are repeated three times for each protein, as shown in Test 1, Test 2, and Test 3. A: MCP-1; B: β-actin; C: CXCL-2; D: β-actin; E: MIP-3α; F: β-actin. The order of samples was CON, LPS, rosavin (16 μM), rosavin (32 μM), rosavin (64 μM).

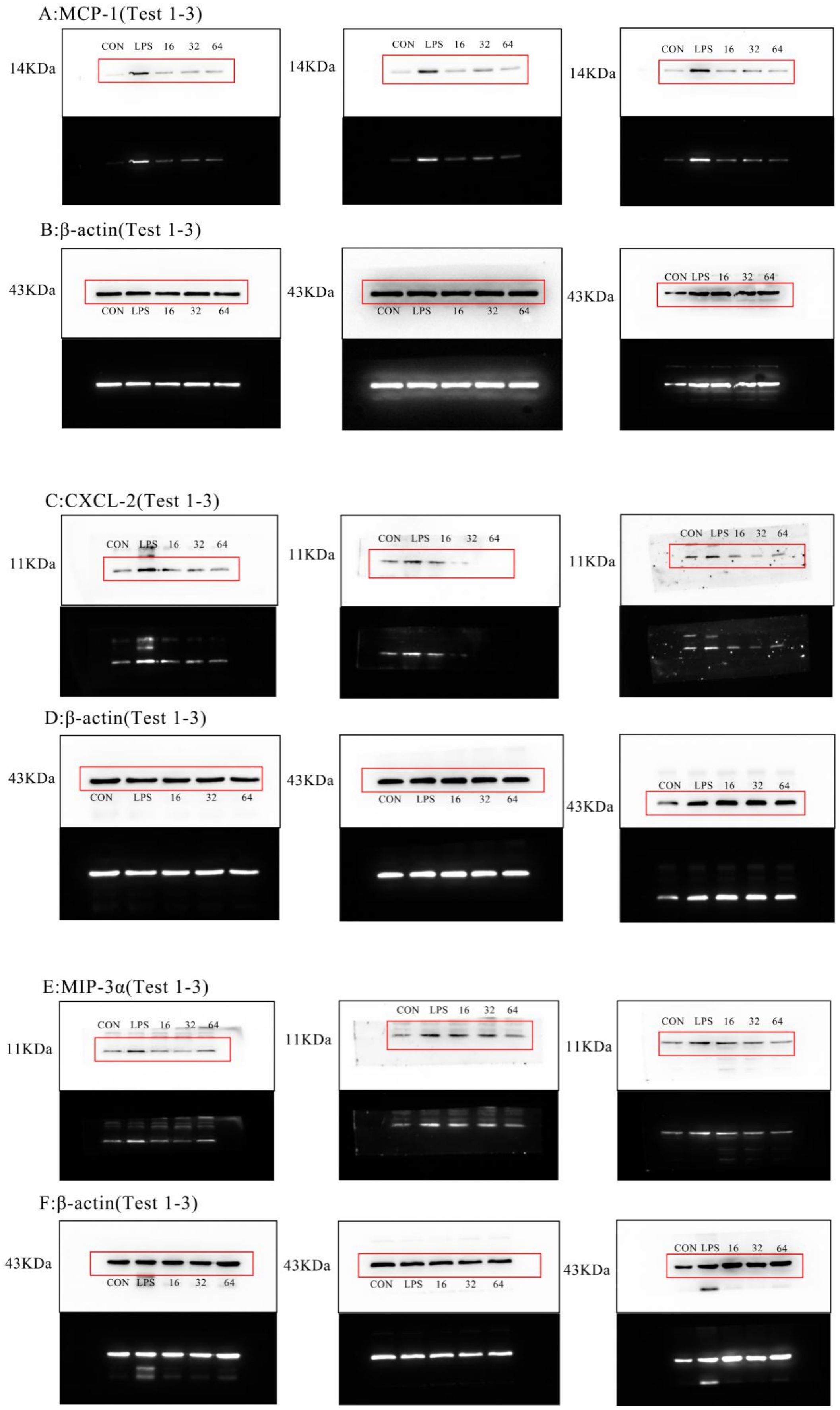
A:MCP-1(Test 1-3)
14KDa
CON LPS 16 32 64
B:β-actin(Test 1-3)
43KDa
CON LPS 16 32 64
C:CXCL-2(Test 1-3)
11KDa
CON LPS 16 32 64
D:β-actin(Test 1-3)
43KDa
CON LPS 16 32 64
E:MIP-3α(Test 1-3)
11KDa
CON LPS 16 32 64
F:β-actin(Test 1-3)
43KDa
CON LPS 16 32 64

**Figure3**. Panels A-D represent the manuscript entitled "Rosavin alleviates LPS-induced acute lung injure by modulating the TLR-4/NF-κB/MAPK singnaling pathways" in figure 5. The entire uncropped images of the original Western blots shown are repeated three times for each protein, as shown in Test 1, Test 2, and Test 3. A: p-ERK; B: ERK; C: p-JNK; D: JNK. The order of samples was CON, LPS, rosavin (16 μM), rosavin (32 μM), rosavin (64 μM).

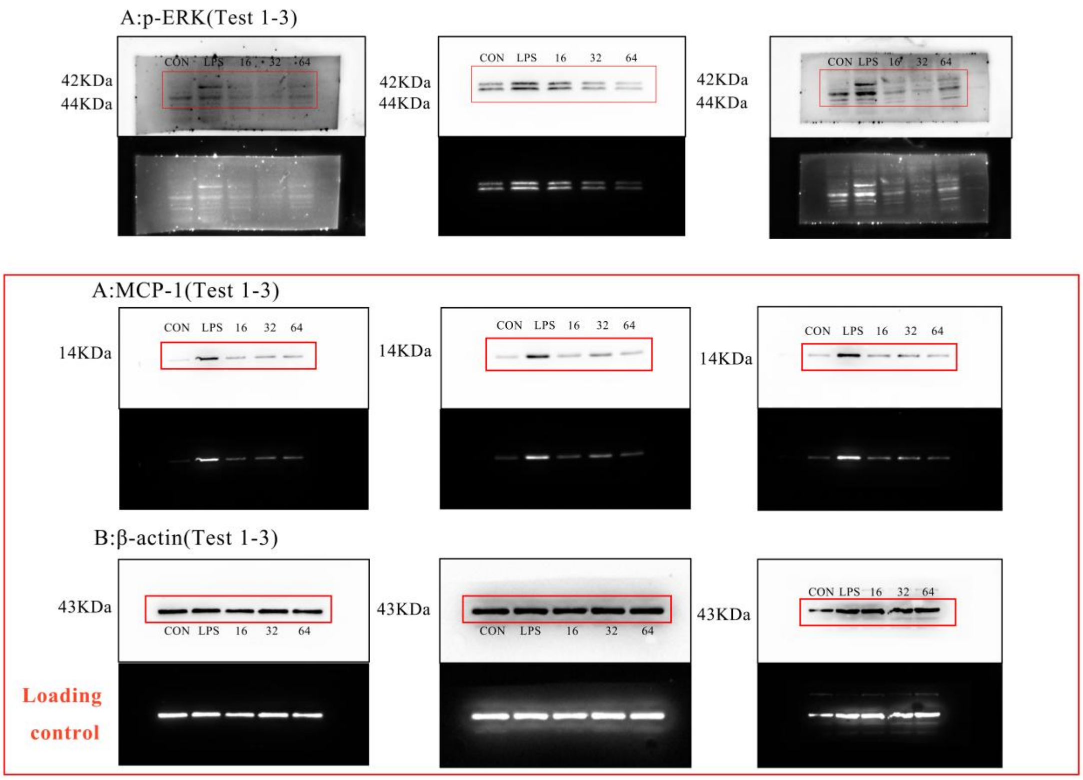

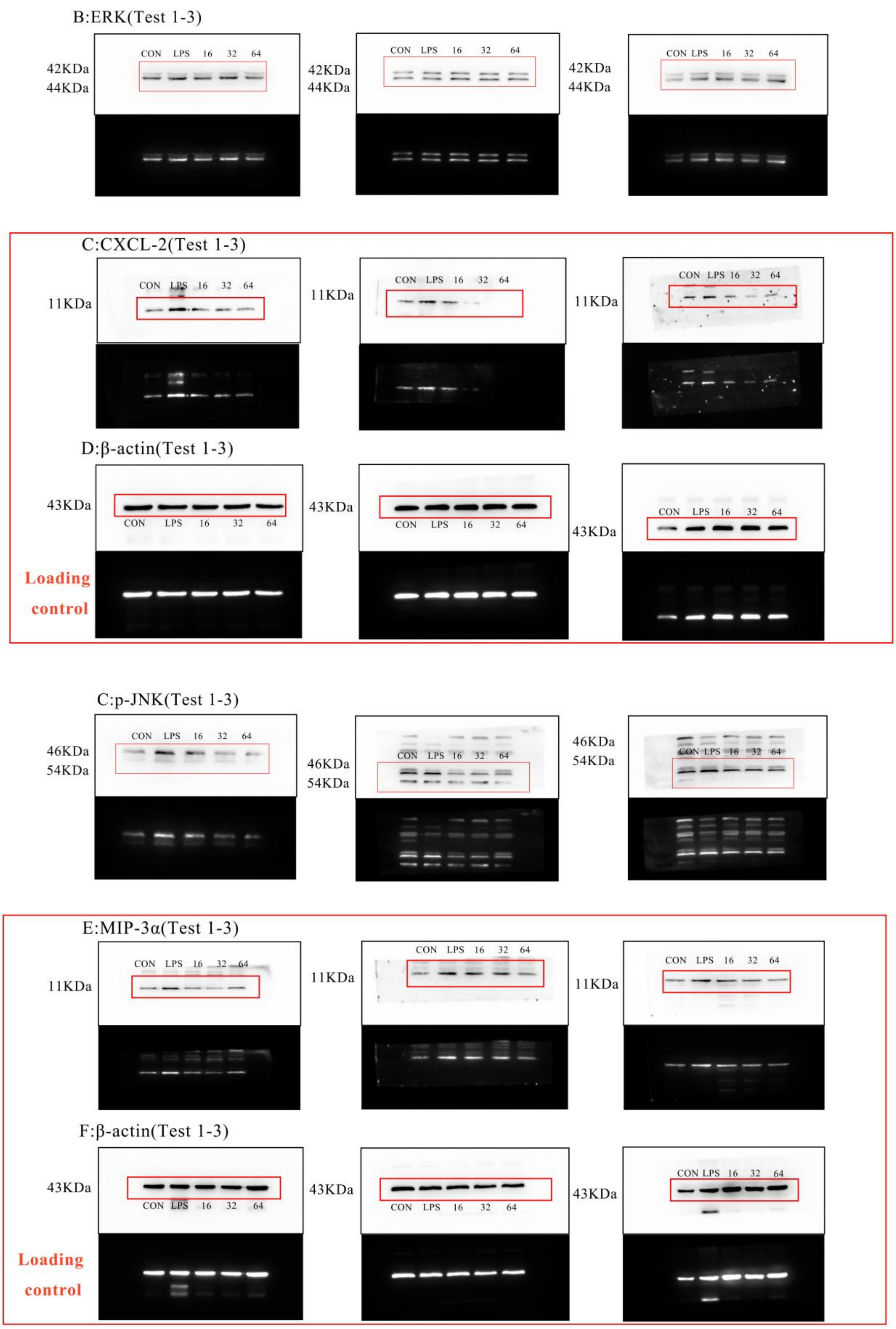

B:ERK(Test 1-3)
42KDa
44KDa
CON LPS 16 32 64
C:CXCL-2(Test 1-3)
11KDa
D:β-actin(Test 1-3)
43KDa
Loading control
C:p-JNK(Test 1-3)
46KDa
54KDa
E:MIP-3α(Test 1-3)
11KDa
F:β-actin(Test 1-3)
43KDa
Loading control

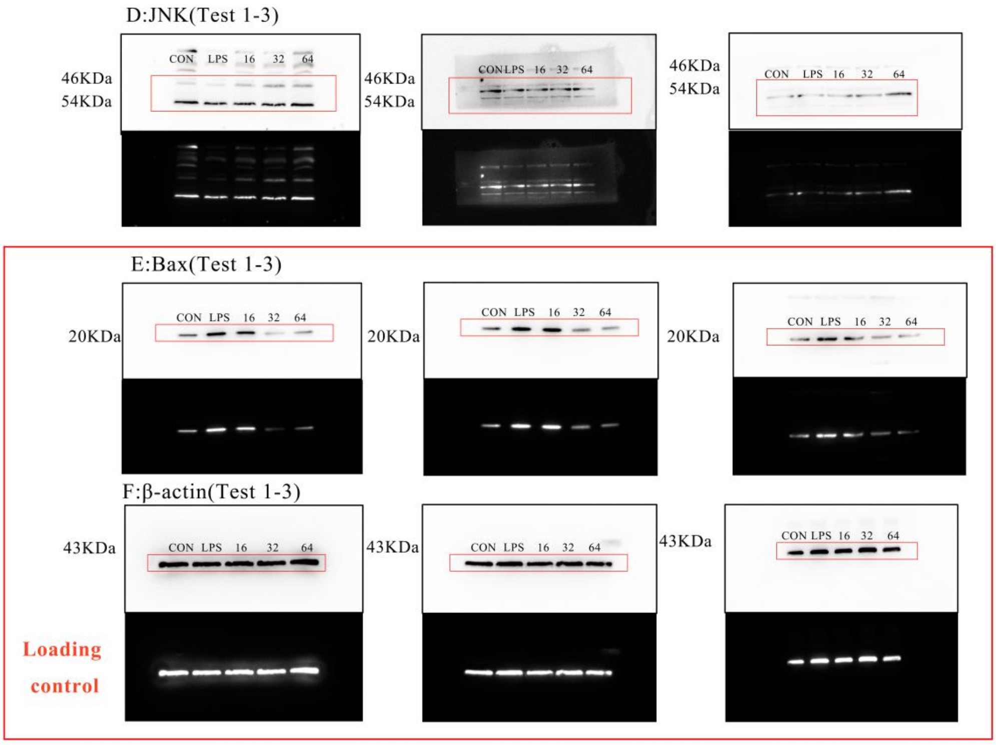


**Figure4**. Panels A-D represent the manuscript entitled "Rosavin alleviates LPS-induced acute lung injure by modulating the TLR-4/NF-κB/MAPK singnaling pathways" in figure 6. The entire uncropped images of the original Western blots shown are repeated three times for each protein, as shown in Test 1, Test 2, and Test 3. A: Bax; B: β-actin; C: Bcl-2; D: β-actin. The order of samples was CON, LPS, rosavin (16 μM), rosavin (32 μM), rosavin (64 μM).

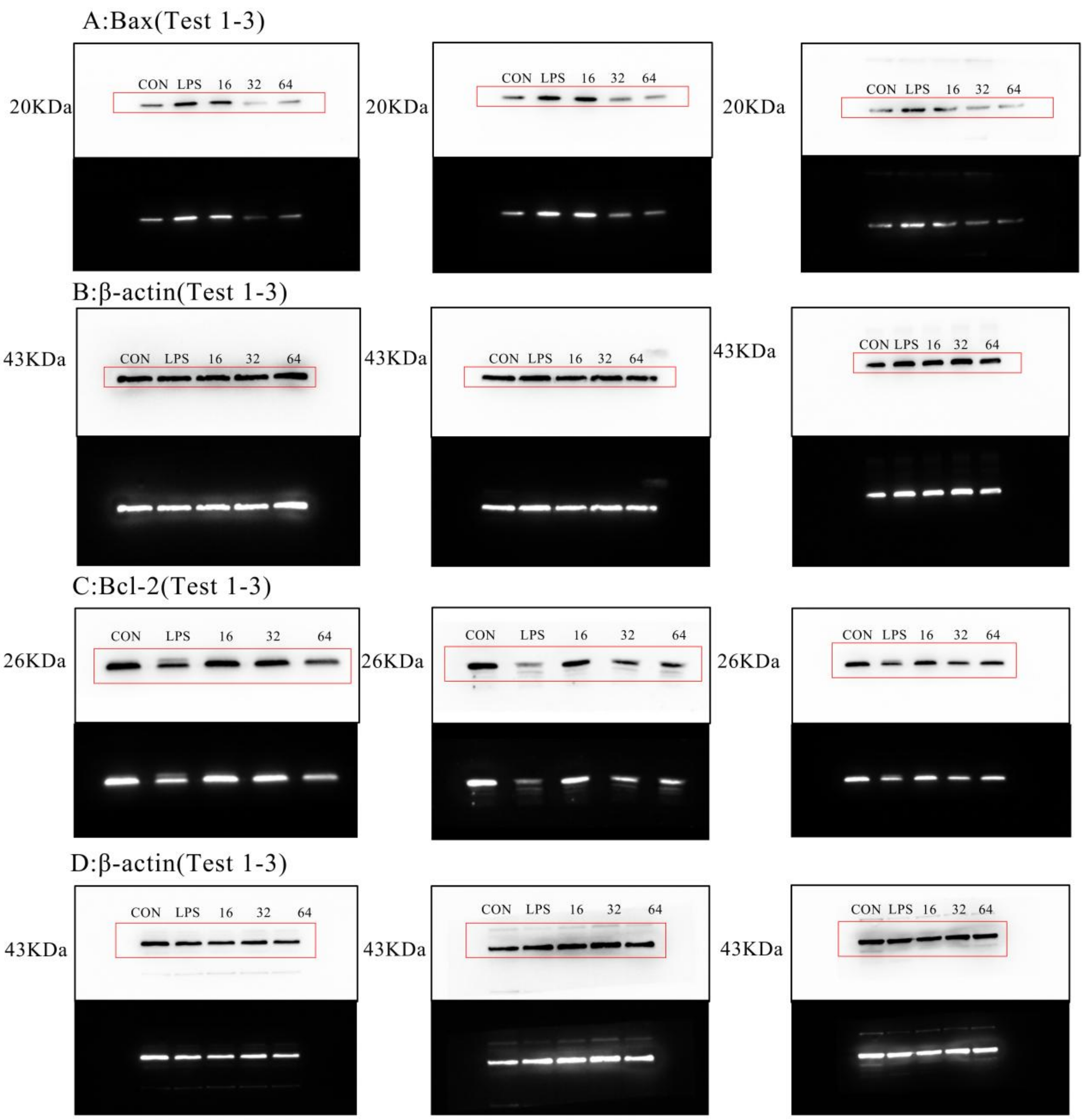


**Figure5**.Panels A-I represent the manuscript entitled "Rosavin alleviates LPS-induced acute lung injure by modulating the TLR-4/NF-κB/MAPK singnaling pathways" in figure 12. The entire uncropped images of the original Western blots shown are repeated three times for each protein, as shown in Test 1, Test 2, and Test 3. A: TLR-4; B: MyD88; C: β-Tubulin; D: iNOS; E: β-actin; F: Nuclear NF-κB p65; G: Histone-H3; H: Cytoplasm NF-κB p65; I: GAPDH. The order of samples was CON, LPS, rosavin (20 mg/kg), rosavin (40 mg/kg), rosavin (80 mg/kg), DEX.

A:TLR-4(Test 1-3)

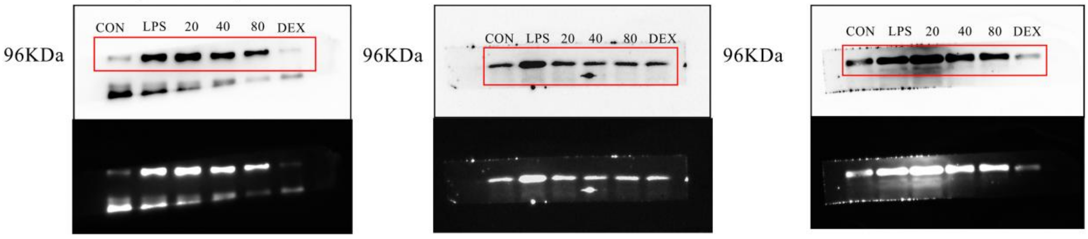


B:MyD88(Test 1-3)

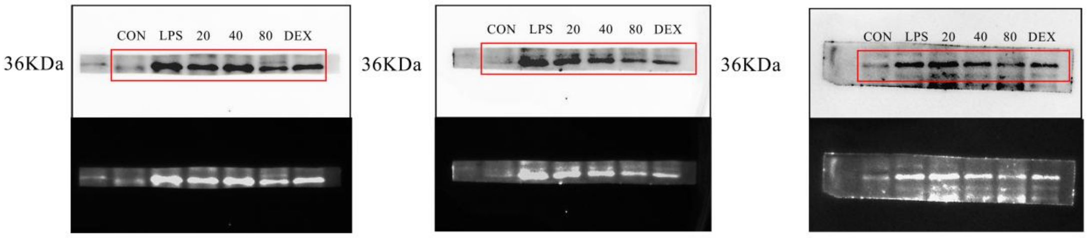


C:β-Tubulin(Test 1-3)

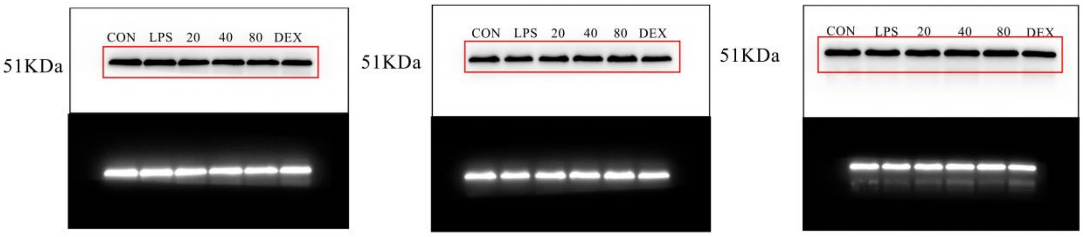

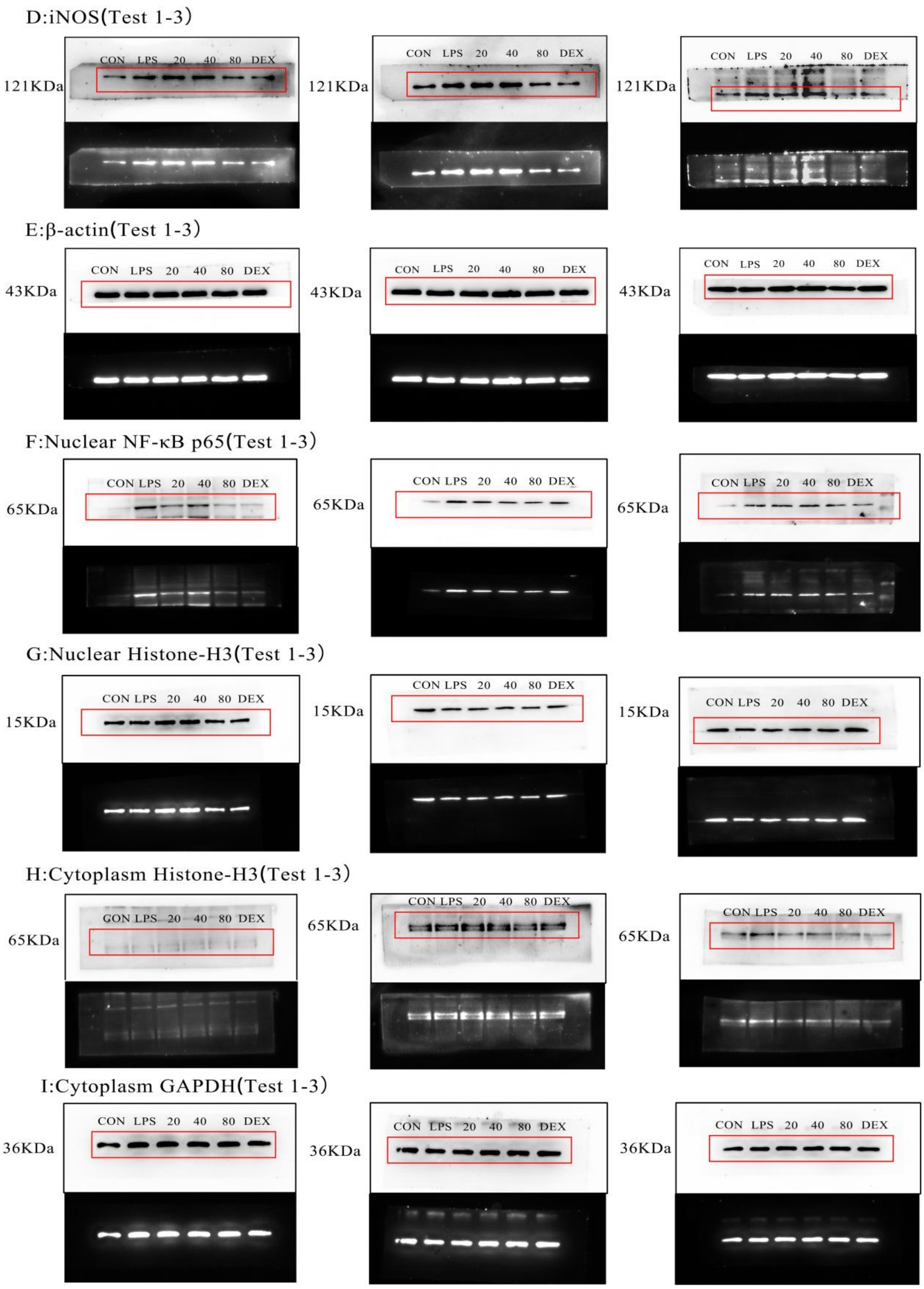


**Figure6**.Panels A-L represent the manuscript entitled "Rosavin alleviates LPS-induced acute lung injure by modulating the TLR-4/NF-κB/MAPK singnaling pathways" in figure 13. The entire uncropped images of the original Western blots

shown are repeated three times for each protein, as shown in Test 1, Test 2, and Test 3. A: p-ERK; B: β-Tubulin; C: ERK; D: β-Tubulin; E: p-p38; F: β-Tubulin; G: p38; H: β-Tubulin; I: p-JNK; J: GAPDH; K: JNK; L: GAPDH. The order of samples was CON, LPS, rosavin (20 mg/kg), rosavin (40 mg/kg), rosavin (80 mg/kg), DEX.

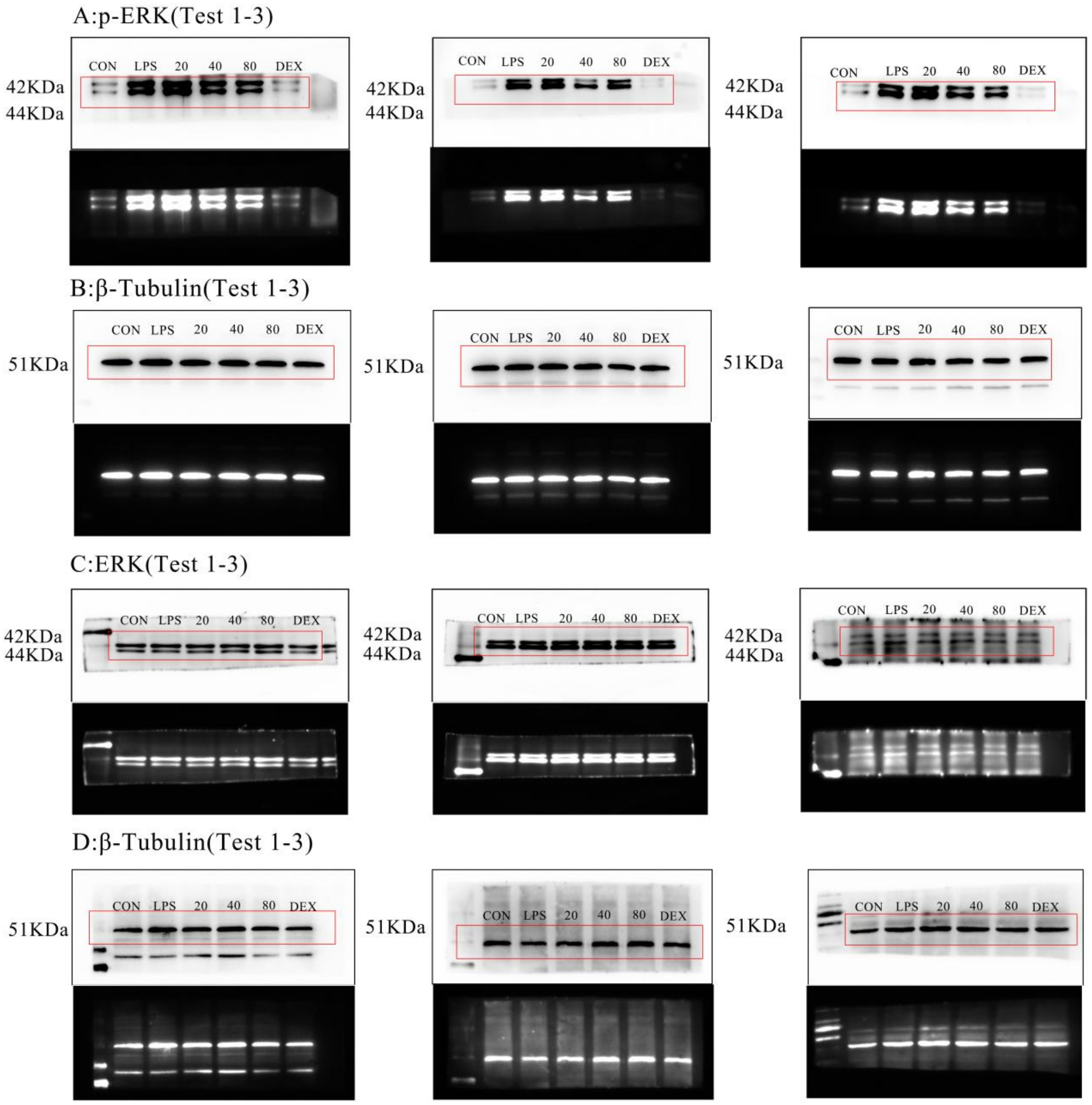

E:p-p38(Test 1-3)

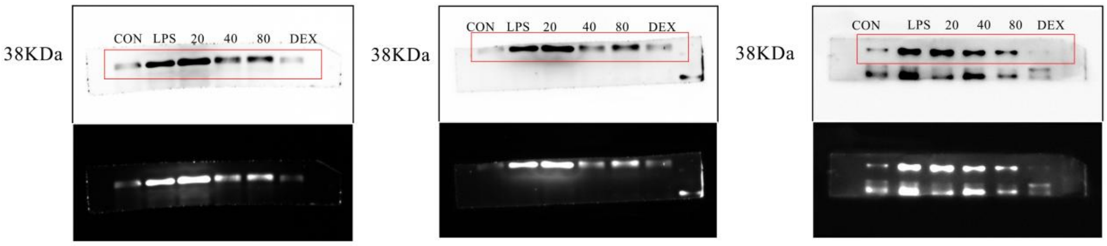


F:β-Tubulin(Test 1-3)

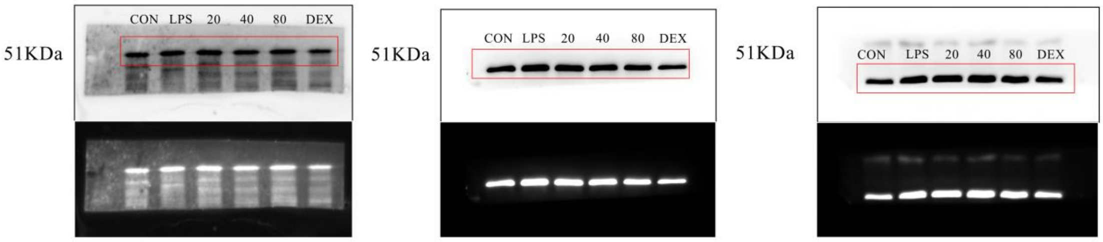


G:p38(Test 1-3)

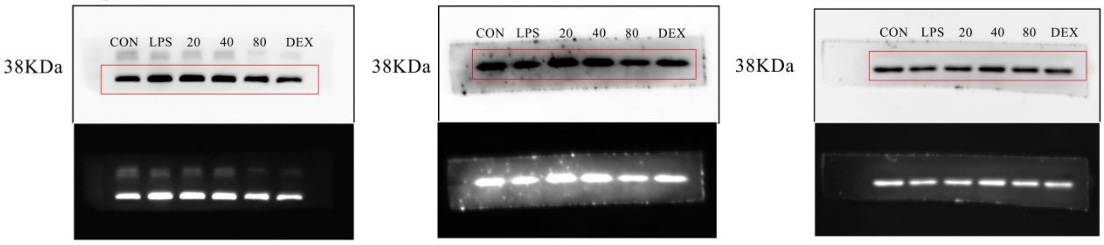


H:β-Tubulin(Test 1-3)

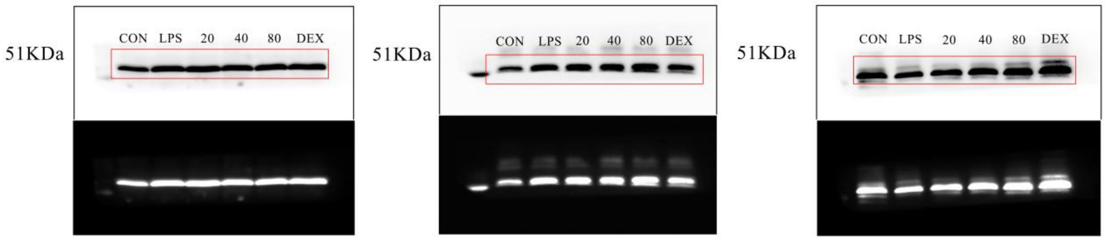

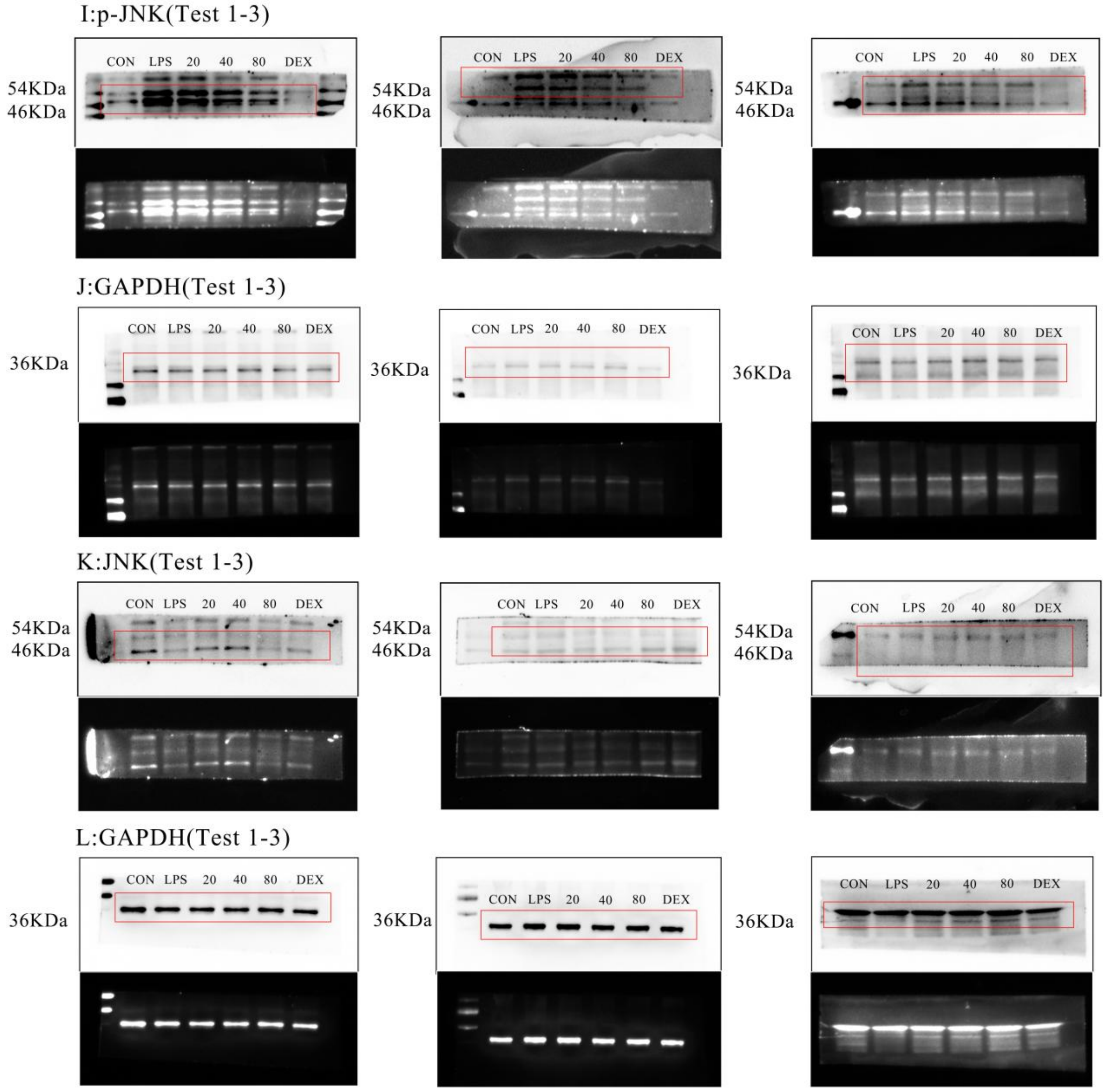


**Figure7**.Panels A-D represent the manuscript entitled "Rosavin alleviates LPS-induced acute lung injure by modulating the TLR-4/NF-κB/MAPK singnaling pathways" in figure 14. The entire uncropped images of the original Western blots shown are repeated three times for each protein, as shown in Test 1, Test 2, and Test 3. A: Bax; B: β-actin; C: Bcl-2; D: β-actin. The order of samples was CON, LPS, rosavin (20 mg/kg), rosavin (40 mg/kg), rosavin (80 mg/kg), DEX.

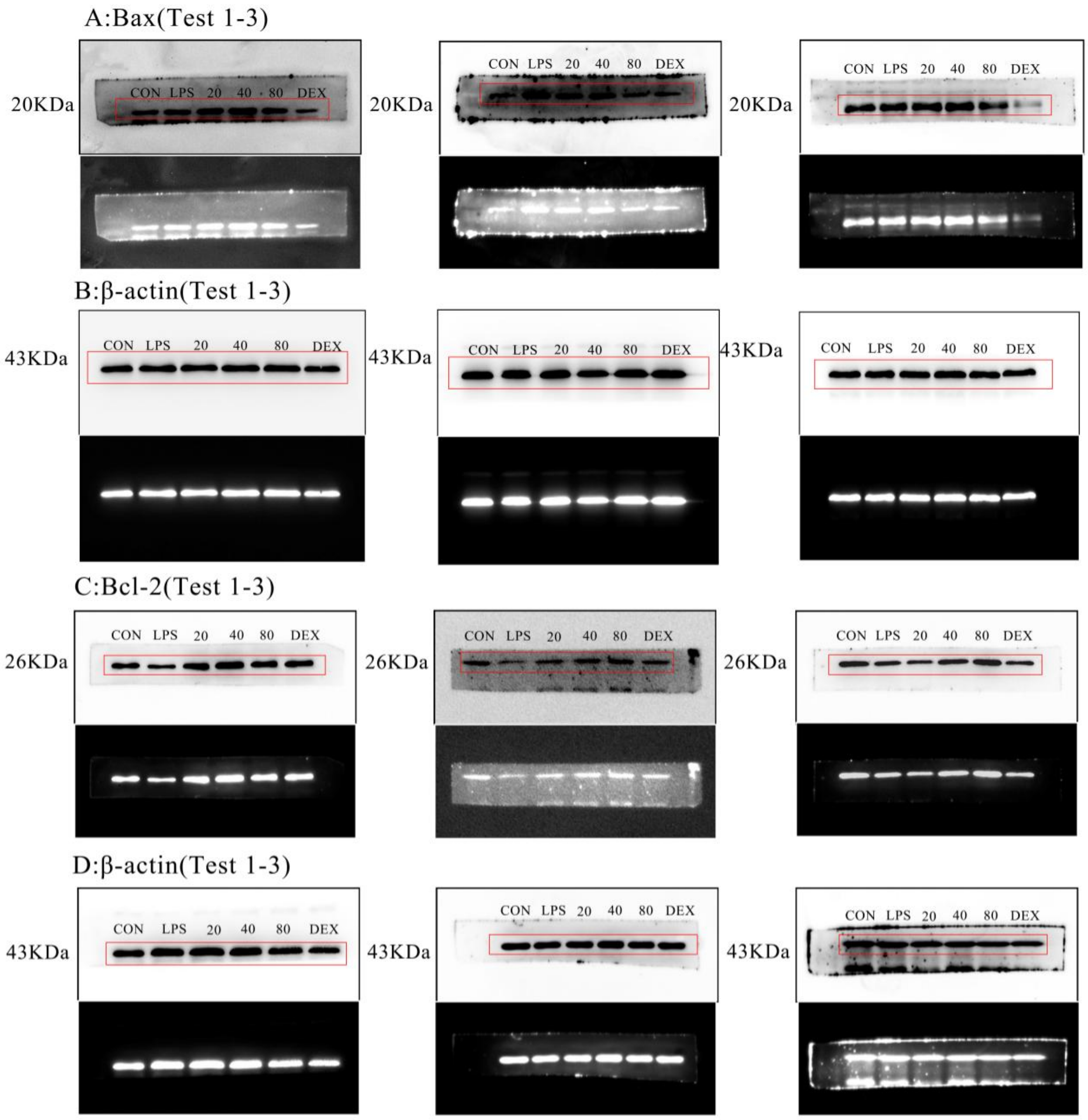
A:Bax(Test 1-3)
20KDa
CON LPS 20 40 80 DEX
B:β-actin(Test 1-3)
43KDa
CON LPS 20 40 80 DEX
C:Bcl-2(Test 1-3)
26KDa
CON LPS 20 40 80 DEX
D:β-actin(Test 1-3)
43KDa
CON LPS 20 40 80 DEX
