# Supplementary material for: Rosavin Alleviates LPS-Induced Acute Lung Injure by Modulating the TLR-4/NF-κB/MAPK Singnaling Pathways
Source: Int J Mol Sci. 2024 Feb 3;25(3):1875. doi: 10.3390/ijms25031875 (PMC10856478; doi:10.3390/ijms25031875)
Supplement: Supplementary file 1 [file ijms-25-01875-s001.zip › Supplementary Material.pdf]

Supplementary Material

Part SI the purity of rosavin

Figure S1. High performance liquid chromatography of rosavin detected at 254 nm.

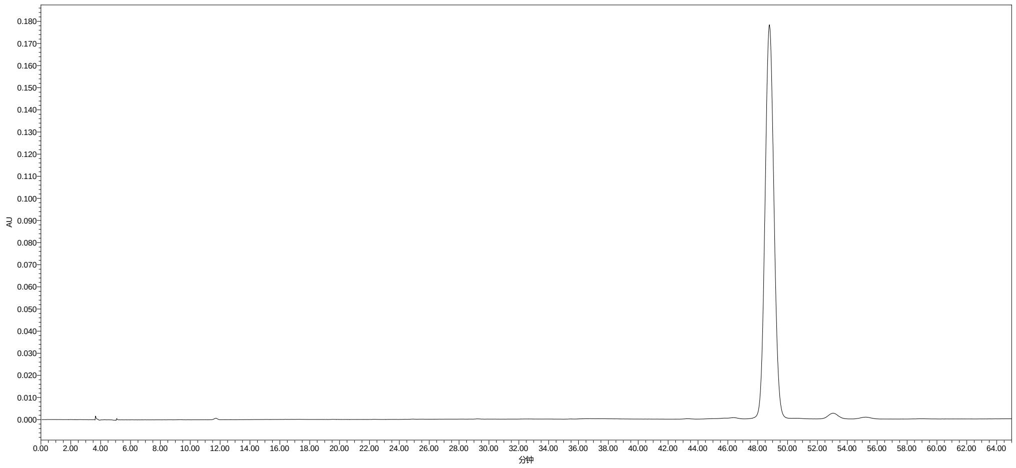

Number	Observerd RT(min)	Peak area(mAu*s)	Area pecentage(%)	Peak Type
1	3.658	9919	0.0013	BB
2	5.077	3083	0.0004	BB
3	11.735	7684	0.0010	BB
4	48.783	7308980	98.5740	BB
5	53.070	86044	0.0116	BB
Total		7415710	100.0000	

Figure S2. 1H NMR spectra of rosavin

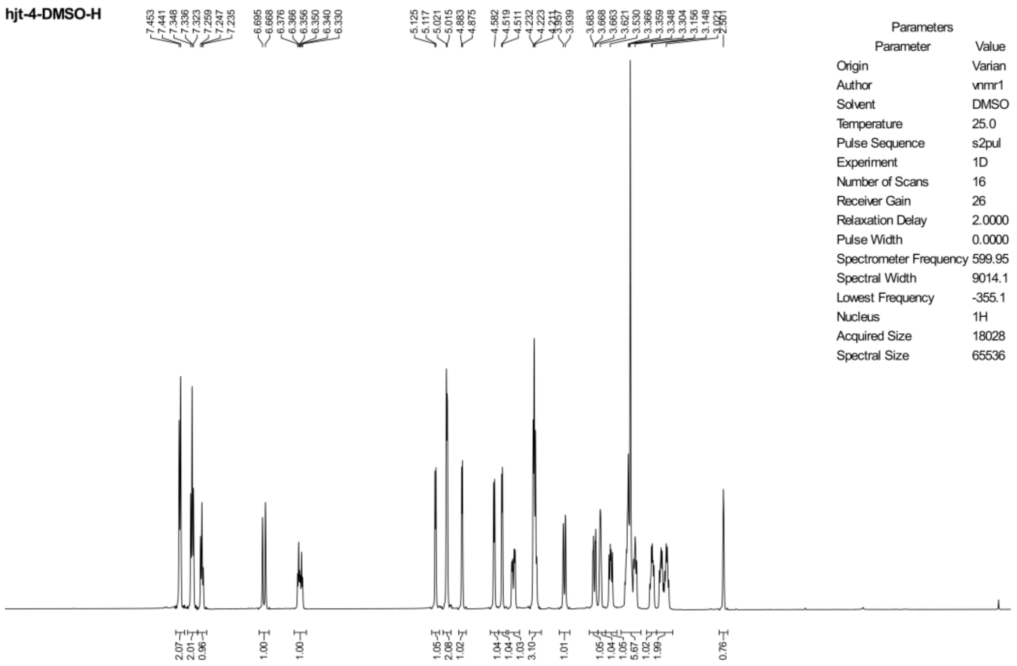

Figure S3. 13C NMR spectra of rosavin

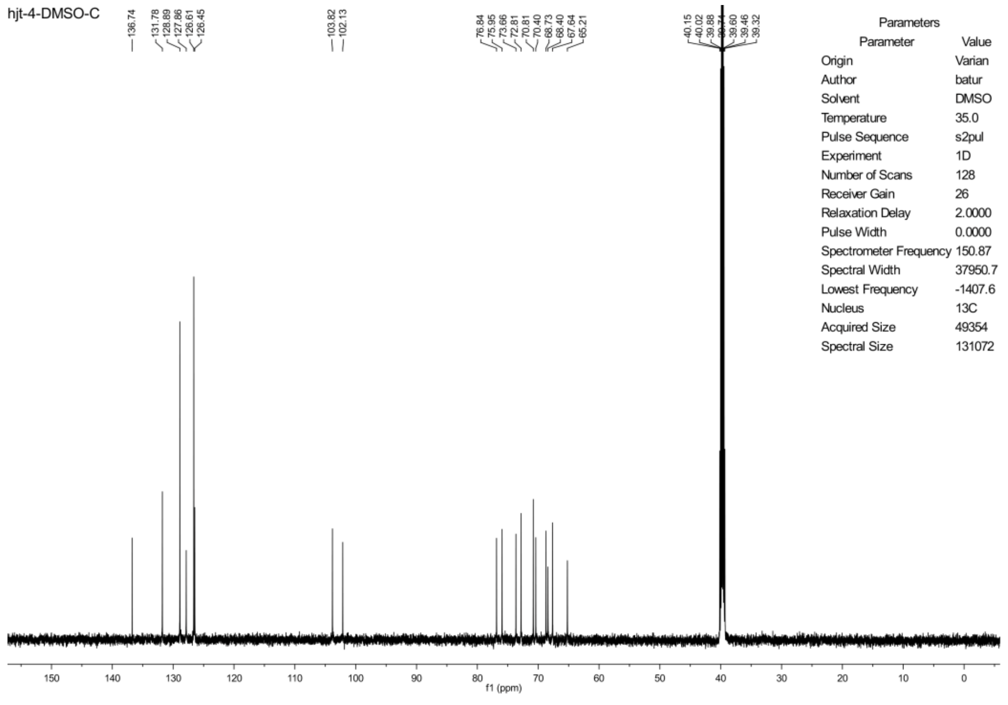

Part SII Notes on the Ethics of Animal Experimentation

石河子大学第一附属医院动物实验伦理审查表

Animal Experimental Ethical Review Form
of the First Affiliated Hospital of Shihezi University

〔2023〕院伦审动实字（149）号(**No.A2023-149-01**)

申请人填写的相关信息 Related Information Filed by Applicant

项目名称 Project Title	Rosvain 通过 TLR4/NF-κB/MAPK 途径减轻 LPS 诱导的急性肺损伤		
项目来源与编号 Funding Source & Number	国家重大专项新药创制项目(编号：2018ZX09735-005)子项目		
课题负责人 Principal Investigator	张珂	科 室 Department	无
职 称 Professional Title	副教授	动物实验岗位证书编号 Animal User Permit No.	无
参与实验人员 Additional Personnel	姓 名 Name	职 称 Professional Title	动物实验岗位证书编号 Animal User Permit No.
	刘巧慧	无	无
	丰姝姝	无	无

使用动物情况 Animal Requirements

动物实验设施许可证编号 Facility License No.	无		
动物来源 Source of animal	新疆医科大学实验动物中心，生产许可证号 SCXK（新）2018-0001		
品种/品系 Species or Strains	Balb/C	等 级 Grade	SPF 级
规 格 Specifications	无	数 量 Number	♂72 只 ♀0 只 共 72 只
计划进驻日期 Proposed Date of Commencement	2021 年 12 月 30 日	计划结束日期 Proposed Date of Completion	2022 年 1 月 30 日
动物实验室主任意见 Director Attitude of Lab Animal Unit	同意 Agree √ 不同意 Disagree □	签名(Signature)：陈文	2023 年 3 月 17 日

实验要点〔包括实验目的，实验方法，观测指标，术前、术中、术后照护（如镇痛），确定人道终点，实验结束后处死动物的方法等〕Outline of experiments〔including aim of experiment, experimental methods, observational index, peri-, intra- and post-operative care（e.g. analgesia）of animals, determination of humane endpoints, and executing animal method, et al〕

建立 LPS 诱导的小鼠肺损伤模型，观察 Rosvain 对肺损伤小鼠的影响，检测小鼠肺组织中 MPO、MDA、SOD 和 GSH-Px 的含量；血清中 IL-1β、TNF-α和 IL-6 水平；TLR4、MYD88、NF-κB 等关键蛋白表达水平，进一步探索 Rosvain 对小鼠肺损伤的保护作用的具体机制，实验过程中，遵循人道的实验终结点，采用腹腔注射 1%戊巴比妥钠溶液、颈椎脱臼的处死方法，减少动物的痛苦、紧迫与不适。

申请者签名 Signature of Applicant	刘巧慧	联系电话 Telephone	办公室(O)	无
			移 动(M)	18892996323

动物伦理审查表

<table>
<tr><td>申请者声明
Declaration of Principal Investigator</td><td colspan="3">我将自觉遵守实验动物福利伦理原则，随时接受实验动物伦理委员会的监督与检查，如违反规定，自愿接受处罚。(I will abide by the rules of animal experimental ethics, accept the supervision and inspection of the animal experimental ethics committee, and accept the punishment if any infringement.)

签名(Signature): [illegible]

2023 年 3 月 17 日</td></tr>
<tr><td>审查依据
Ethical Considerations</td><td colspan="3">1. 该项目是否必须用实验动物进行实验，即能否用计算机模拟、细胞培养等非生命方法替代动物或用低等动物替代高等动物进行实验(Does laboratory animal must be used in the project? Could other methods such as computer simulation，cell culture or using the low-grade animal instead of the high－grade animal?)

2. 表中所填申请人资格和所用动物的品种品系、质量等级、规格是否合适，能否通过改良设计方案或用高质量的动物来减少所用动物的数量(Are the qualification of applicant，species or strain，grade and specifications of animals suitable? Could the quantity of animals be reduced by improving the study design or using high quality animals?)

3. 能否通过改进实验方法、调整实验观测指标、给予必要的术后照护（如镇痛）、改良处死动物的方法，来优化实验方案、善待动物(Could the study design and animal treatment be refined by ameliorating experimental method，adjusting observational index，providing necessary post-operative care（e.g. analgesia），or executing animal method?)</td></tr>
<tr><td>医学伦理委员会意见
MEC</td><td>同意
Agree ☑</td><td>不同意
Disagree ☐</td><td rowspan="2">伦理委员会主任/授权者签字
Chair of Medical Ethics Committee
[illegible]

签章(Stamp):
2023 年 3 月 22 日</td></tr>
<tr><td>备注
Remark</td><td colspan="2"></td></tr>
</table>
